# Functional characterization of squalene synthase and squalene epoxidase in *Taraxacum koksaghyz*

**DOI:** 10.1002/pld3.63

**Published:** 2018-06-13

**Authors:** Kristina Unland, Katharina M. Pütter, Kirsten Vorwerk, Nicole van Deenen, Richard M. Twyman, Dirk Prüfer, Christian Schulze Gronover

**Affiliations:** ^1^ Fraunhofer Institute for Molecular Biology and Applied Ecology (IME) Muenster Germany; ^2^ Institute of Plant Biology and Biotechnology University of Muenster Muenster Germany; ^3^ TRM Ltd Scarborough UK

**Keywords:** latex, oxidosqualene cyclase, pentacyclic triterpene, RNA interference, squalene epoxidase, squalene synthase, *Taraxacum koksaghyz* (Russian dandelion), transcriptional regulation

## Abstract

The Russian dandelion *Taraxacum koksaghyz* produces high‐value isoprenoids such as pentacyclic triterpenes and natural rubber in the latex of specialized cells known as laticifers. Squalene synthase (SQS) and squalene epoxidase (SQE) catalyze key steps in the biosynthesis of cyclic terpenoids, but neither enzyme has yet been characterized in *T. koksaghyz*. Genomic analysis revealed the presence of two genes (*TkSQS1* and *TkSQS2*) encoding isoforms of SQS, and four genes (*TkSQE1–4*) encoding isoforms of *SQE*. Spatial expression analysis in different *T. koksaghyz* tissues confirmed that TkSQS1 and TkSQE1 are the latex‐predominant isoforms, with highly similar mRNA expression profiles. The TkSQS1 and TkSQE1 proteins colocalized in the endoplasmic reticulum membrane and their enzymatic functions were confirmed by in vitro activity assays and yeast complementation studies, respectively. The functions of TkSQS1 and TkSQE1 were further characterized in the latex of *T. koksaghyz* plants with depleted *TkSQS1* or *TkSQE1* mRNA levels, produced by RNA interference. Comprehensive expression analysis revealed the coregulation of *TkSQS1* and *TkSQE1*, along with a downstream gene in the triterpene biosynthesis pathway encoding the oxidosqualene cyclase TkOSC1. This indicates that the coregulation of *TkSQS1*,* TkSQE1,* and *TkOSC1* could be used to optimize the flux toward specific terpenoids during development.

## 1 INTRODUCTION

The dandelion species *Taraxacum koksaghyz* (Tk) produces a plethora of secondary metabolites in specialized cells known as laticifers, including high‐value isoprenoids such as the polyisoprene natural rubber, sterols, and pentacyclic triterpenes. The pentacyclic triterpenes in particular exhibit extraordinary biological activities against fungi and bacteria, making them ideal for agricultural and pharmaceutical applications (Ghosh, 2016).

**Figure 1:**
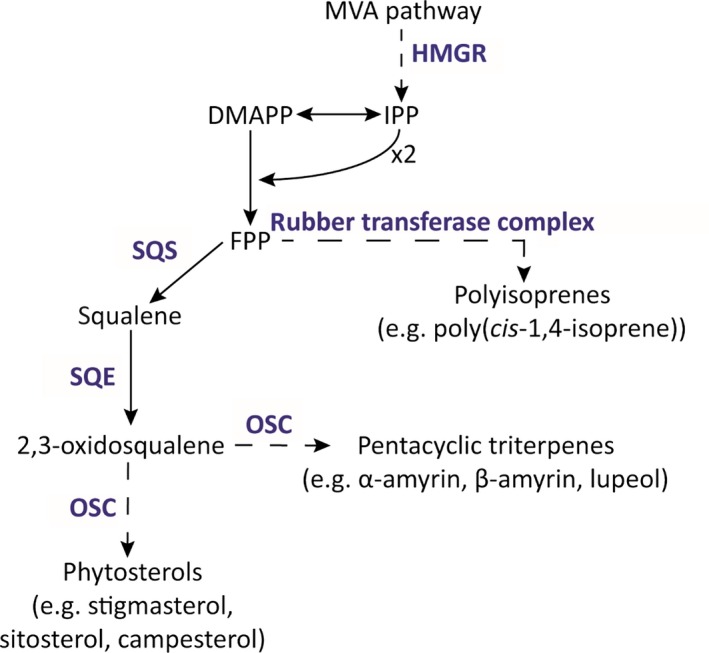
Proposed isoprenoid biosynthesis pathway in *Taraxacum koksaghyz* latex. Enzymes or enzyme complexes are shown in blue, and dashed arrows indicate multiple enzymatic steps. DMAPP, dimethylallyl diphosphate; FPP, farnesyl diphosphate; HMGR, 3‐hydroxy‐methyl‐glutaryl‐CoA reductase; IPP, isopentenyl diphosphate; MVA, mevalonate; OSC, oxidosqualene cyclase; SQE, squalene epoxidase; SQS, squalene synthase

The biosynthesis of pentacyclic triterpenes requires squalene synthase (SQS, EC 2.5.1.21) and squalene epoxidase (SQE, EC 1.14.14.7) as rate‐limiting enzymes. SQS is a bifunctional membrane‐bound enzyme that catalyzes the synthesis of squalene from two C_15_ allylic farnesyl diphosphate (FPP) molecules in two steps. First, presqualene diphosphate is produced via the head‐to‐head condensation of two FPP molecules, and this is subsequently reduced to squalene in an NADPH‐dependent second step requiring divalent cations (Jarstfer, Zhang, & Poulter, 2002). Only a single *SQS* gene is found in yeast and humans (Jennings, Tsay, Fisch, & Robinson, 1991; Robinson, Tsay, Kienzle, Smith‐Monroy, & Bishop, 1993), whereas there are 1–3 *SQS* genes in plants. Thus far, single *SQS* genes have been reported among others in *Oryza sativa* (Hata et al., 1997), *Lotus japonicus* (Akamine et al., 2003), *Taxus cuspidata* (Huang et al., 2007), and *Euphorbia tirucalli* (Uchida et al., 2009), whereas two paralogs are found in *Nicotiana tabacum* (Devarenne, Shin, Back, Yin, & Chappell, 1998), *Glycyrrhiza glabra* (Hayashi, Hirota, Hiraoka, & Ikeshiro, 1999), *Glycine max* (Nguyen et al., 2013), *Malus domestica* (Navarro Gallón et al., 2017), and *Arabidopsis thaliana*, although in the last case, one copy was shown to be a pseudogene (Busquets et al., 2008). *Panax ginseng* possesses three *SQS* paralogs (Kim, Han, Huh, & Choi, 2011). SQS enzymes contain a C‐terminal hydrophobic transmembrane domain that anchors the enzyme into the endoplasmic reticulum (ER) membrane, whereas the large catalytic N‐terminal domain is located in the cytosol (Stamellos et al., 1993). Because squalene is the first precursor of triterpenoids such as sterols, brassinosteroids, and pentacyclic triterpenes, SQS activity is an important switch and major branching point between triterpene and polyisoprene biosynthesis (Figure 1). As such, SQS represents a rate‐limiting step in triterpene biosynthesis and is associated with the overall yield of these secondary metabolites. This has been demonstrated in several plant species: the overexpression of *SQS* in *P. ginseng* and *Eleutherococcus senticosus* increased the overall SQS activity and caused the accumulation of sterols and triterpenes (Lee et al., 2004; Seo et al., 2005), whereas the virus‐induced gene silencing of *SQS* in *Withania somnifera* and *M. domestica* caused the sterol levels to decline (Navarro Gallón et al., 2017; Singh et al., 2015). Various RNA interference (RNAi) methods have also been used to silence the *SQS* gene and reduce sterol levels and this also caused the accumulation of artemisinin in *Artemisia annua* (Zhang et al., 2009). Moreover, tobacco cell suspension cultures treated with a fungal elicitor revealed that SQS is regulated at multiple transcriptional and post‐translational levels (Devarenne, Ghosh, & Chappell, 2002; Devarenne et al., 1998).

Squalene epoxidase (SQE) catalyzes the epoxidation of squalene to 2,3‐oxidosqualene, which is the first oxidation reaction in the triterpene biosynthesis pathway. Subsequently, 2,3‐oxidosqualene is converted into various triterpene end‐products by oxidosqualene cyclases (OSCs). The oxidation of squalene requires O_2_ as well as NADPH and FAD cofactors (Abe & Prestwich, 1999; Nakamura & Sato, 1979; Ono, Ozasa, Hasegawa, & Imai, 1977). In *Saccharomyces cerevisiae*, SQE is located in the ER and is associated with lipid particles (Leber et al., 1998). In plants, SQE is anchored to the ER via transmembrane domains, although the number of predicted transmembrane domains can vary within a species (Han, In, Kwon, & Choi, 2010; He, Zhu, He, & Zhang, 2008; Laranjeira et al., 2015). Like *SQS*, only single *SQE* genes are present in yeast and humans, whereas some plants possess multiple paralogs, for example, *P. ginseng* and *A. thaliana* (Han et al., 2010; Rasbery et al., 2007). The different isoforms encoded by these genes may fulfill different functions; for example, only three of the six SQE isoforms in *A. thaliana* restore wild‐type activity to the yeast *erg1* mutant. And among them, only *AtSQE1* is essential for growth and development, given that *Atsqe1* mutant plants suffer from defects such as impaired stem elongation and infertility (Rasbery et al., 2007). In *P. ginseng*, two SQE isoforms with different regulatory roles were identified by treatment with methyl jasmonate, which suppresses *PgSQE2* expression but induces the accumulation of *PgSQE1* mRNA in roots. In addition, the knockdown of *PgSQE1* reduced the level of ginsenosides, whereas the overexpression of *PgSQE2* and *PNX* (encoding cycloartenol synthase) increased the accumulation of sterols (Han et al., 2010).

To gain deeper insight into the roles of SQS and SQE in the regulation of isoprenoid biosynthesis in *T. koksaghyz* latex, the isoforms of both enzymes were identified and functionally characterized *in planta*. The transcriptional regulation of the corresponding genes was analyzed at different developmental stages in wild‐type dandelion plants and in *SQS* and *SQE* RNAi lines.

## 2 MATERIALS AND METHODS

### 2.1 Plant material and cultivation conditions

*Taraxacum koksaghyz* wild‐type and transgenic plants were cultivated at 18°C and 20 klux (high pressure sodium lamp, HPS 600 W, Greenbud, enhanced yellow and red spectrum) with a 16‐h photoperiod in controlled growth chambers or in the greenhouse. Plants were cultivated in a pre‐fertilized 1:1 mixture of standard soil (ED73 Einheitserde, Fröndenberg, Germany) and garden mold (Botanical Garden Münster, Germany). They were fed every 4 weeks with a commercial fertilizer according to the manufacturer's recommendations (Hakaphos Plus, Compo GmbH, Münster, Germany). *Nicotiana benthamiana* seeds were obtained from the Sainsbury Laboratory (John Innes Centre, Norwich, UK) and cultivated as stated above.

### 2.2 Total RNA extraction and cDNA synthesis

Total RNA was extracted from *T. koksaghyz* latex, root, leaf, peduncle and flower tissues using the innuPREP RNA Mini Kit (Analytik Jena, Jena, Germany) according to the manufacturer's instructions. Full‐length cDNA was synthesized from 500 ng total RNA using PrimeScript RT Master Mix (TaKaRa, Clontech, Saint‐Germain‐en‐Laye, France) according to the manufacturer's instructions.

### 2.3 In silico analysis

*TkSQS1* and *TkSQE1‐2* full‐length cDNA sequences were obtained from RNA‐Seq data, whereas *TkSQS2* and *TkSQE3‐4* full‐length gDNA sequences were identified from draft genome data (Lin et al., 2017).

Full‐length cDNA was translated using the ExPASy translate tool (http://web.expasy.org/translate/). The isoelectric point and molecular weight were predicted using the ProtParam tool (http://web.expasy.org/protparam/), and protein secondary structures were predicted using SOPMA (Geourjon & Deleage, 1995; https://npsa-prabi.ibcp.fr/cgi-bin/npsa_automat.pl?page=/NPSA/npsa_sopma.html). Conserved protein domains were identified using the NCBI conserved domain search (https://www.ncbi.nlm.nih.gov/Structure/cdd/wrpsb.cgi) and PROSITE (http://prosite.expasy.org/), whereas transmembrane domains were predicted using TMHMM software (http://www.cbs.dtu.dk/services/TMHMM-2.0/).

### 2.4 Phylogenetic analysis

Amino acid sequences were obtained from GenBank (https://www.ncbi.nlm.nih.gov/genbank/) and are listed in Supporting Information Table S1. Multiple alignments of protein sequences were generated using the Clustal MUSCLE algorithm (Edgar, 2004; http://www.ebi.ac.uk/Tools/msa/muscle/). Phylogenetic trees were created using MEGA6 (Tamura, Stecher, Peterson, Filipski, & Kumar, 2013; http://www.megasoftware.net/).

### 2.5 Quantitative RT‐PCR

Quantitative RT‐PCR analysis was carried out as previously described (Pütter, van Deenen, Unland, Prüfer, & Schulze Gronover, 2017). *Taraxacum koksaghyz* wild‐type plants were grown for 12 weeks for spatial expression analysis and for 8–20 weeks for temporal expression analysis. RNA was extracted from nine individual plants, before pooling the cDNA from three plants. All oligonucleotide sequences for expression analysis are listed in Supporting Information Table S2. Primer efficiencies and amplification factors are summarized in Supporting Information Table S3.

### 2.6 Isolation of genomic DNA

Genomic DNA was isolated using the NucleoSpin Plant II Kit (Macherey‐Nagel, Düren, Germany) according to the manufacturer's instructions.

### 2.7 Cloning and stable transformation procedures

For localization studies, the full‐length *TkSQS1* cDNA was amplified using primers TkSQS1‐fwd‐blunt and TkSQS1‐rev‐XhoI, digested with XhoI and inserted into the XmnI/XhoI sites of the Gateway‐compatible vector pENTR3C (Thermo Fisher Scientific Inc., Darmstadt, Germany), resulting in the final vector pENTR3C‐TkSQS1. The full‐length *TkSQE1* cDNA was amplified using primers TkSQE1‐fwd‐NcoI and TkSQE1‐rev‐NotI, and a similar fragment lacking the stop codon (*TkSQE1‐wos*) was amplified using the primers TkSQE1‐fwd‐NcoI and TkSQE1‐wos‐rev‐NotI. Both fragments were digested with NcoI and NotI and inserted into the NcoI/NotI sites of the Gateway‐compatible vector pENTR4 (Thermo Fisher Scientific). For localization analysis, the full‐length *TkSQS1* and *TkSQE1* sequences were introduced into the Gateway‐compatible vector pBatTL‐Cerulean‐ccdB, whereas the *TkSQE1‐wos* fragment was introduced into the vector pBatTL‐ccdB‐Cerulean (Epping et al., 2015) by LR recombination using LR Clonase (Thermo Fisher Scientific). The pBatTL‐Cerulean‐ccdB vector was cloned by amplifying the Cerulean fragment from a pUC18 Cerulean vector (GenBank: CAO79587) using the primer combination Cerulean‐BglII‐fwd and Cerulean‐BglII‐rev, digesting the plasmid with the corresponding restriction enzyme and inserting the product into the BglII site of pBaTL‐ccdB (Jach, Pesch, Richter, Frings, & Uhrig, 2006).

For TkSQS1 activity assays, the *TkSQS1* full‐length sequence was introduced into the Gateway‐compatible vector pBatTL‐ccdb to yield pBatTL‐TkSQS1. The latter was subsequently introduced into *Agrobacterium tumefaciens* strain GV3103pMP90RK as previously described (Pütter et al., 2017).

For the TkSQE1 complementation assay, pENTR4‐TkSQE1 was introduced into the Gateway‐compatible destination vectors and pAG425GPD‐ccdB‐HA (Addgene, Cambridge, MA, USA). The expression vector pLab12.5‐pREF containing the promoter of the rubber elongation factor (REF) was prepared as previously described (Epping et al., 2015). For the construction of pLab12.5‐pREF‐TkSQS1‐RNAi and pLab12.5‐pREF‐TkSQE1‐RNAi, a 178‐bp TkSQS1‐RNAi and a 285‐bp TkSQE1‐RNAi PCR fragment were amplified using the RNA dicer‐optimized primers TkSQS1‐RNAi‐fwd‐NcoI and TkSQS1‐RNAi‐rev‐XhoI or TkSQE1‐RNAi‐fwd‐NcoI and TkSQE1‐RNAi‐rev‐XhoI, respectively. Both RNAi fragments were inserted into the NcoI and XhoI sites of the Gateway vector pBluescript II KS (+) (Addgene). Subsequently, the TkSQS1‐RNAi and the TkSQE1‐RNAi fragments were transferred to the pLab12.5‐pREF vector by LR recombination to generate pLab12.5‐pREF‐TkSQS1‐RNAi and pLab12.5‐pREF‐TkSQE1‐RNAi. The transformation of *T. koksaghyz* by *A. tumefaciens* strain EHA105 was carried out as previously described (Stolze et al., 2017). Oligonucleotide sequences are shown in Supporting Information Table S4.

### 2.8 Methyl jasmonate assay

*Taraxacum koksaghyz* wild‐type plants were cultivated under standard conditions for 6 weeks, subsequently the plants were repotted into cylindrical vessels (diameter 3.5 cm, height 5 cm) in vermiculite (Nestaan, Blandain, Belgium). Eight‐week‐old plants were watered with 25 ml of 0.8 mmol/L MeJA (Sigma‐Aldrich, St. Louis, USA) or water as previously described (Cao et al., 2017). As MeJA (95%) was dissolved in absolute ethanol before dilution, the same amount of ethanol was added to the water used for the control plants. Six hours after the treatment latex was harvested from three plants each and used for mRNA expression analysis.

### 2.9 Subcellular localization studies

Subcellular localization studies were carried out as previously described (Epping et al., 2015).

*In silico* analysis revealed the presence of a single TkSQS1 C‐terminal transmembrane domain, so an N‐terminal fusion with the blue fluorescent protein Cerulean (Cerulean‐TkSQS1) was used for the localization studies. In contrast, TkSQE1 was shown to contain both N‐terminal and C‐terminal transmembrane domains, so we prepared and tested both N‐terminal and C‐terminal fusions (Cerulean‐TkSQE1 and TkSQE1‐Cerulean). All three fusion proteins were transiently expressed in *N. benthamiana* leaf cells, which were analyzed by confocal laser scanning microscopy (CLSM). We used the monomeric red fluorescent protein (mRFP) as a cytosolic marker and the N‐terminal sequence of CYP51G1 fused to mRFP (NtermCYP51G1‐mRFP) as an ER marker, to facilitate the localization of the Cerulean fusion proteins. NtermCYP51G1‐mRFP presents mRFP on the cytosolic surface of the ER (Bassard, Mutterer, Duval, & Werck‐Reichhart, 2012).

For TkSQS1 and TkSQE1 localization, the corresponding pBatTL constructs were infiltrated into *N. benthamiana* leaves in the following combinations: pBatTL‐Cerulean‐TkSQS1 +  pBatTL‐mRFP, pBatTL‐Cerulean‐TkSQS1 +  pBatTL‐NtermCYP51G1‐mRFP, pBatTL‐TkSQE1‐Cerulean + pBatTL‐mRFP, pBatTL‐TkSQE1‐Cerulean+ pBatTL‐NtermCYP51G1‐mRFP, pBatTL‐Cerulean + pBatTL‐NtermCYP51G1‐mRFP.

### 2.10 SQS activity assay

*Nicotiana benthamiana* leaves were infiltrated with the binary vector pBatTL‐TkSQS1 in which *TkSQS1* was controlled by the constitutive Cauliflower mosaic virus 35*S* promoter. After incubation for 5 days, a crude protein extract from leaves was tested using an in vitro SQS assay with [1,2‐^14^C]‐labeled FPP as the substrate. Protein extraction and quantification were carried out as previously described (Pütter et al., 2017). SQS activity was assessed in vitro followed by reversed‐phase thin‐layer chromatography analysis and quantification by scintillation counting as described by Busquets et al. (2008).

### 2.11 Yeast complementation assay

For the yeast complementation assay of SQE activity, the *S. cerevisiae* (Δ*erg1*) mutant stain KLN1 (*MAT (*α*), ERG1::URA3, leu2, ura3, trp1*) (Landl, Klönsch, & Turnowsky, 1996) and KLN1‐pERG1 (a KLN1 derivative carrying a leucine‐selectable expression plasmid for *ERG1*) were kindly provided by *F. Turnowsky* (University of Graz, Austria). KLN1 was transformed with the vector pAG425GPD‐TkSQE1 or the empty vector by lithium acetate transformation as previously described (Hill, Donald & Griffiths, 1991). A drop test was performed on SD‐Leu minimal medium under aerobic conditions and under anaerobic conditions with the supplement of 12 μg/μl ergosterol and 0.5% (v/v) Tween‐80. The yeast were suspended in Tris‐EDTA buffer, dropped out at OD_600 nm_ 1, OD_600 nm_ 0.1, OD_600 nm_ 0.01, and OD_600 nm_ 0.001 and incubated for 3 days under anaerobic conditions or 5 days under aerobic conditions.

### 2.12 Chemical analysis

Whole *T. koksaghyz* roots were frozen in liquid nitrogen, freeze‐dried, and crushed to a fine powder. For triterpene analysis, 100 mg of dry root material was used for saponification by adding 20 ml of methanol containing 6% potassium hydroxide and heating to 80°C for 2 hr. As an internal standard, we added 100 μl of betuline (2.5 mg/ml stock solution in acetone). Samples were extracted three times with 1 volume of hexane. The hexane phases were pooled and evaporated, and the samples were redissolved in 1 ml of acetone. Triterpene analysis was performed with a GC‐MS‐QP 210 Ultra system (Shimadzu, Duisburg, Germany) equipped with a Rxi^®^‐5 ms column (Restek GmbH, Bad Homburg, Germany). We injected 0.5 μl of the extract using split modus (1:10) at an injector and interface temperature of 260°C. The GC temperature program was as follows: 120°C for 3 min, temperature gradient of 15°C per minute up to 330°C, 330°C for 10 min. Electron ionization (EI) in the MS was set to 70 eV. Peak integration and identification was performed with the LabSolution software (Shimadzu, Duisburg, Germany) using a NIST library (NIST = National Institute of Standards and Technology) or by analyzing the corresponding standards obtained from Extrasynthese (Genay, France). Quantification was performed in relation to the internal standard. Retention indices for the triterpene compounds were determined using the LabSolution software in relation to a C8–C40 alkane calibration standard (Sigma‐Aldrich, Taufkirchen, Germany) using the same operating conditions.

## 3 RESULTS

### 3.1 Molecular cloning and sequence analysis of two *SQS* paralogs (*TkSQS1* and *TkSQS2*) and four *SQE* paralogs (*TkSQE1–TkSQE4*) in *T. koksaghyz*

RNA‐Seq data obtained from latex, root, and leaf mRNA expression profiles and the recently published draft genome of *T. koksaghyz* (Lin et al., 2017) were screened for the presence of *SQS* and *SQE* cDNA sequences. One contig showed high similarity to known *SQS* sequences and was strongly expressed in *T. koksaghyz* latex. The primers TkSQS1‐fwd and TkSQS1‐rev were therefore designed to amplify the full‐length cDNA sequence, subsequently designated *TkSQS1*. The resulting 1257‐bp cDNA was predicted to encode a protein of 418 amino acids (GenBank: MG646369). A second SQS gene (*TkSQS2*) in Contig6767 was identified in the draft genome sequence, and the full‐length cDNA was obtained from peduncle mRNA by PCR using primers TkSQS2‐fwd and TkSQS2‐rev. The 1254‐bp cDNA was predicted to encode a protein of 417 amino acids (GenBank: MG646370). The TkSQS1 and TkSQS2 proteins had predicted isoelectric points of 8.18 and 8.57, respectively. The calculated molecular weights were 47.981 and 47.713 kDa, respectively. The secondary structure of TkSQS1 was predicted to consist mainly of α‐helices (65%), followed by random coils (18%), extended strands (9%), and β‐turns (8%), and was therefore quite similar to that of TkSQS2, with 60% α‐helices, 22% random coils, 11% extended strands, and 7% β‐turns. The TkSQS1 and TkSQS2 amino acid sequences were aligned in silico with known SQS proteins from other plant species (Supporting Information Figure S1a). TkSQS1 showed high identity to SQS from *Helianthus annuus* (91%) and *A. annua* (89%), with lower sequence identity to SQS from *Hevea brasiliensis* (81%), *N. tabacum* (80%), *A. thaliana* (78%), and *Zea mays* (70%). Interestingly, TkSQS1 showed only 72% sequence identity to TkSQS2, which in turn showed comparable identities to the SQS proteins from *H. annuus*,* H. brasiliensis* and *A. annua* (73%, 72% and 71%, respectively). TkSQS2 showed only 64% sequence identity to *Z. mays* SQS.

**Figure 2:**
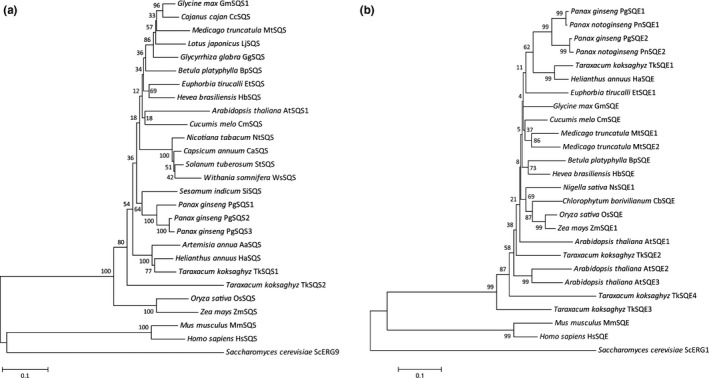
Phylogenetic tree of (a) SQS and (b) SQE amino acid sequences. The tree was constructed using the neighbor‐joining method in MEGA6 software (Tamura et al., 2013; http://www.megasoftware.net/) with a bootstrap of 1000 replicates. Protein sequences were obtained from GenBank (https://www.ncbi.nlm.nih.gov/genbank/) and are provided in Table S1

TkSQS1 and TkSQS2 belong to the isoprenoid biosynthesis enzymes class 1 superfamily (Conserved Protein Domain Family Accession cl00210) and possess three conserved SQS domains that are typical for mammalian SQS proteins and are known to be involved in catalysis (Supporting Information Figure S1a; Gu, Ishii, Spencer, & Shechter, 1998). Domain A, with its conserved tyrosine residue Y171, is presumably responsible for the first step of catalysis and is identical to the squalene and phytoene synthases signature 1 pattern (PROSITE Accession Number PS01044) at TkSQS1 residues 168–183 and TkSQS2 residues 169–184. For TkSQS1, domain B encompasses residues 199–226 and contains the essential conserved amino acids D213 and D217 involved in substrate binding via the magnesium salt bridge, whereas in TkSQS2, domain B encompasses residues 200–227 with D214 and D218 as the conserved amino acids (Gu et al., 1998). This domain also includes the characteristic signature 2 pattern of squalene and phytoene synthases (PROSITE Accession Number PS01045), which spans TkSQS1 amino acid residues 201–221 and TkSQS2 amino acid residues 202–222. Domain C is responsible for the second catalytic step (TkSQS1 residues 280–297 and TkSQS2 residues 281–298) with the conserved residues P280, F283, F285, and Q290 in TkSQS1, and P281, F284, F286, and Q291 in TkSQS2. All three essential catalytic domains, including the conserved residues responsible for enzyme activity, were present in TkSQS1 and TkSQS2, indicating that both *T. koksaghyz* SQS isoforms may be involved in squalene biosynthesis via the proposed mechanism. In addition, the kingdom‐specific SQS hinge region was identified in all the plant sequences we analyzed (Supporting Information Figure S1a; Linscott, Niehaus, Zhuang, Bell, & Chappell, 2016) and a phylogenetic tree was constructed with *S. cerevisiae* SQS as the outgroup (Figure 2). Computer‐based predictions of transmembrane domains revealed that TkSQS1 and TkSQS2 each possess one C‐terminal transmembrane domain (encompassing amino acid residues 386–408 and 390–407, respectively), which are highly similar to those predicted for *H. annuus* and *A. annua* SQS (Supporting Information Figure S1a). Functionally, this transmembrane domain may anchor the enzyme into the ER membrane (Busquets et al., 2008; Linscott et al., 2016). Interestingly, a second transmembrane domain close to domain C has been predicted in *A. annua*,* M. truncatula,* and *H. brasiliensis* SQS, and identified in *P. ginseng* PgSS1, PgSS2, and PgSS3, as well as *G. max* SQS1 and SQS2, but this was not present in TkSQS1 or TkSQS2 (Kim et al., 2011; Nguyen et al., 2013).

The RNA‐Seq data were also mined for *SQE* sequences, revealing two contigs with significant similarities to *SQE* genes. The specific primer pairs TkSQE1‐fwd/TkSQE1‐rev and TkSQE2‐fwd/TkSQE2‐rev were designed and used to amplify the corresponding cDNA sequences (*TkSQE1* and *TkSQE2*). The *TkSQE1* and *TkSQE2* cDNAs were 1596 and 1554 bp in length, respectively (GenBank: MG646371, MG646372), potentially encoding proteins of 513 (TkSQE1) and 517 (TkSQE2) amino acids. Two further *SQE* genes (*TkSQE3* and *TkSQE4*) were identified in the draft genome and were amplified using the primer combinations TKSQE3‐fwd/TkSQE3‐rev and TKSQE4‐fwd/TkSQE4‐rev. The full‐length 1857‐bp *TkSQE3* cDNA (GenBank: MG646373) potentially encodes a protein of 618 amino acids, whereas the full‐length 1566‐bp *TkSQE4* cDNA (GenBank: MG646374) potentially encodes a protein of 521 amino acids. The predicted isoelectric points of TkSQE1–4 were 8.81, 8.93, 9.39, and 8.88, respectively, and the calculated molecular weights were 58.080, 56.399, 68.107, and 56.550 kDa, respectively. The secondary structure of all four TkSQE proteins predominantly consisted of random coils (31%–37%) and α‐helices (27%–32%) followed by extended strands (24%–27%) and β‐turns (10%–12%). The in silico alignment of the four TkSQE polypeptide sequences revealed a maximum of 72% identity (TkSQE1 vs. TkSQE2) and a minimum of 62% (TkSQE3 vs. TkSQE4) (Supporting Information Figure S1b). TkSQE1, TkSQE3, and TkSQE4 showed the highest sequence identities to *H. annuus* SQE (89%, 69%, and 66%, respectively), whereas TkSQE2 showed greater identity to SQE1 and SQE2 from *P. ginseng* (77% in each case) compared to *H. annuus* SQE (75%). The phylogenic tree showed that TkSQE1–TkSQE4 cluster with diverse SQEs from other plants rather than together and that TkSQE3 shows the longest separation distance from other plant SQEs, indicating a putative functional difference (Figure 2). The *T. koksaghyz* SQEs also possess two conserved domains involved in enzyme activity (motif I) and substrate binding (motif II) that have already been identified in *S. cerevisiae* ERG1 (Uchida et al., 2015). Motif I, also termed the FAD I “fingerprint,” consists of a Rossmann fold with alternating β‐sheets and α‐helices containing the sequence profile GXGXXG (TkSQE1–4 residues 73–78, 60–65, 150–155, and 64–69, respectively), which is needed to bind the ADP moiety of FAD (Ruckenstuhl, Eidenberger, Lang, & Turnowsky, 2005). In addition, the FAD II “fingerprint” contains the conserved GD motif (TkSQE1–4 residues 352–353, 339–340, 429–430, and 343–344, respectively), which binds NAD(P)H and the ribose moiety of FAD (Eppink, Schreuder, & van Berkel, 1997). Both domains are fully conserved in all *T. koksaghyz* SQE sequences (Supporting Information Figure S1b). Furthermore, the prediction of the transmembrane domains revealed that TkSQE1 contains four transmembrane domains, only one is present in TkSQE2, TkSQE3 contains six, and TkSQE4 contains two. The in silico data are summarized in Table S5. Finally, the *TkSQS1*‐*2* and *TkSQE1*‐*4* exon–intron structures were determined (Supporting Information Figure S2). The ubiquitous intron motif GT‐AG was present in all sequences.

### 3.2 Spatial and temporal expression profiles reveal the coregulation of *TkSQS1* and *TkSQE1* in dandelion latex

**Figure 3:**
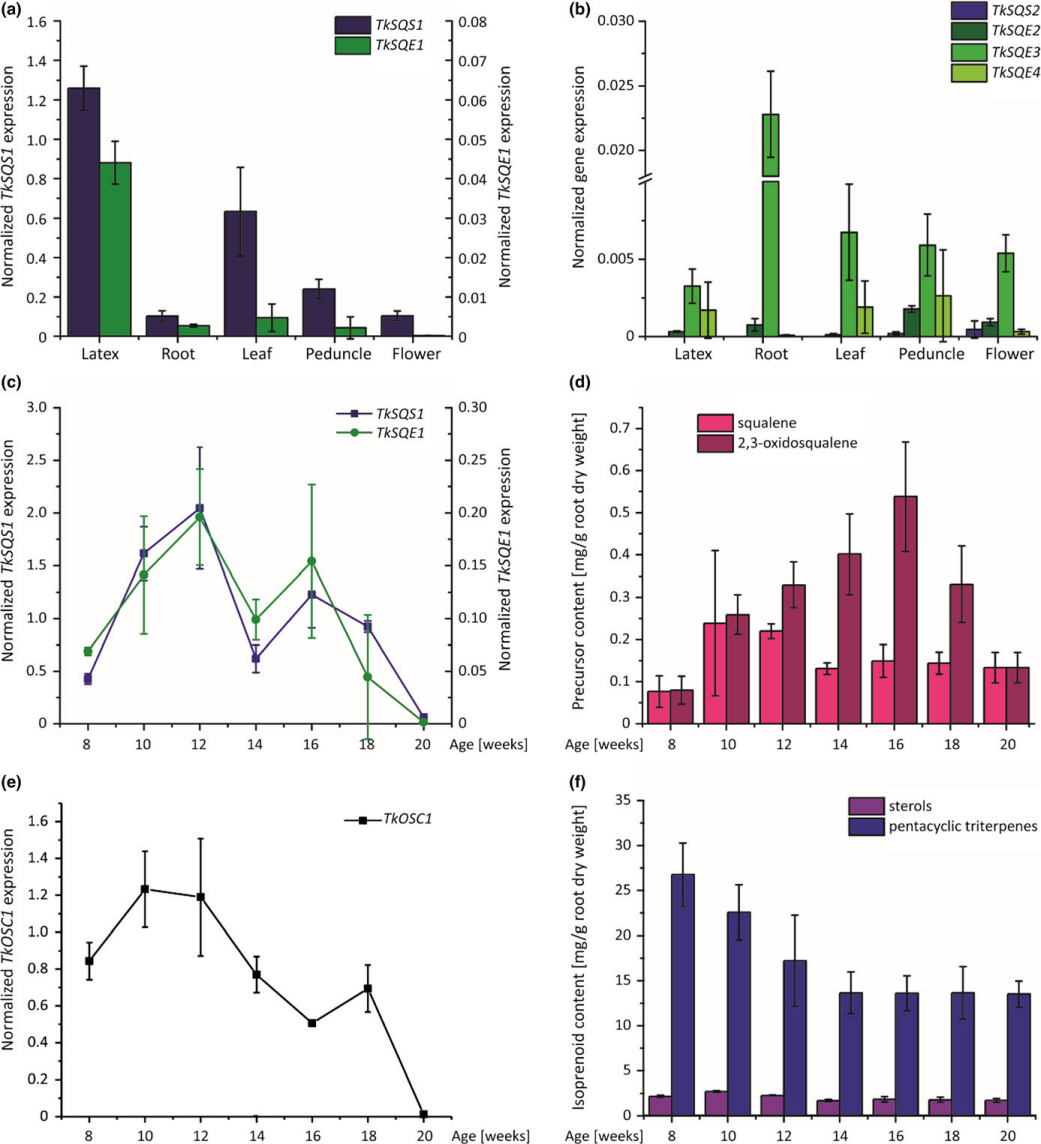
Spatial and temporal expression profiles of *TkSQS*,* TkSQE* and *TkOSC1* mRNA in wild‐type *Taraxacum koksaghyz* plants determined by qRT‐PCR and temporal isoprenoid levels in wild‐type *T. koksaghyz* plants. The corresponding mRNA levels were normalized against the constitutive genes encoding elongation factor 1α (*TkEF1*α) and ribosomal protein L27 (*TkRP*) from *T. koksaghyz*. Data are means ± standard errors of three pools, each consisting of three independent wild‐type plants. (a) *TkSQS1* and *TkSQE1* and (b) *TkSQS2* and *TkSQE2*–*4* mRNA levels in latex, roots, leaves, peduncles, and flowers of 12‐week‐old wild‐type *T. koksaghyz* plants. (c) *TkSQS1* and *TkSQE1* mRNA levels in latex of 8‐ to 20‐week‐old wild‐type *T. koksaghyz* plants. (d) Squalene and 2,3‐oxidosqualene levels in roots of 8‐ to 20‐week‐old *T. koksaghyz* plants. (e) *TkOSC1* mRNA levels in the latex of 8‐ to 20‐week‐old wild‐type *T. koksaghyz* plants. (f) Sterol and pentacyclic triterpene levels in roots of 8‐ to 20‐week‐old wild‐type *T. koksaghyz* plants

The spatial expression patterns of all *TkSQS* and *TkSQE* genes was determined by quantitative real time PCR (qRT‐PCR) using total RNA from the latex, roots, leaves, peduncles and flowers of 12‐week‐old *T. koksaghyz* plants grown under greenhouse conditions. *TkSQS1* was preferentially expressed in the latex, with > threefold higher expression levels compared to leaves and 10‐fold higher expression levels compared to peduncles. Only low levels of *TkSQS1* mRNA were detected in the roots and flowers (Figure 3a). In contrast, *TkSQS2* mRNA was not detected in the latex, roots or leaves, and only low levels were detected in the peduncles and flowers (Figure 3b). TkSQS1 therefore appears to be the key enzyme responsible for isoprenoid biosynthesis in the latex. *TkSQE1* was predominantly expressed in the latex, whereas only low or very low mRNA levels were detected in the leaves, roots, peduncles and flowers (Figure 3a). *TkSQE2*,* TkSQE3,* and *TkSQE4* were expressed at very low levels, particularly in the latex. *TkSQE2* expression was highest in peduncles, whereas *TkSQE3* was expressed most strongly in the roots and *TkSQE4* was expressed at similar levels in the latex, leaves, and peduncles (Figure 3b).

**Figure 4:**
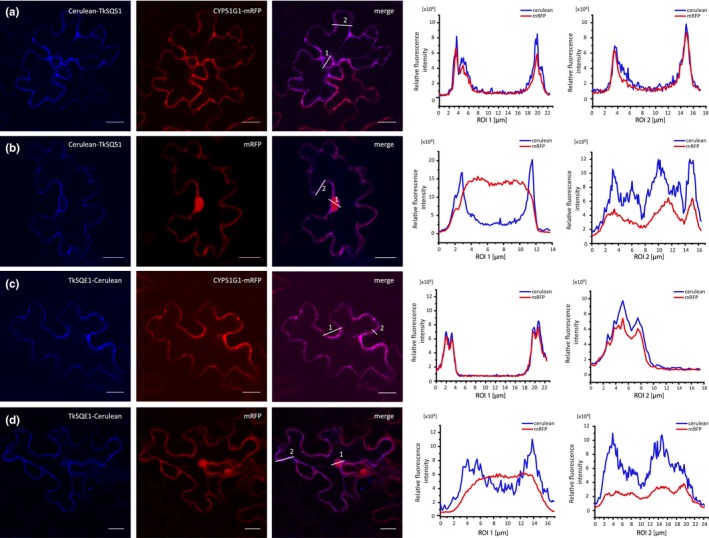
Localization of TkSQS1 and TkSQE1. (a–d) The CLSM images show red (mRFP, excitation 543 nm) and blue (Cerulean, excitation 458 nm) fluorescence of fusion proteins expressed in *Nicotiana benthamiana* epidermal cells. The relative fluorescence intensity in each region of interest (ROI) is marked in two regions. NtermCYP51G1‐mRFP = ER‐localized marker containing a 120‐bp cDNA fragment representing the N‐terminus of *Arabidopsis thaliana* CYP51G1 sterol 14‐demethylase; mRFP = cytosolic marker; scale bar = 20 μm

These results suggest that *TkSQS1* and *TkSQE1* are probably the only genes encoding key enzymes for the biosynthesis of cyclic terpenoids in latex, given the negligible expression of *TkSQS2* and *TkSQE2–TkSQE4* in this specialized cytoplasm. *TkSQS2* and *TkSQE2–TkSQE4* were therefore excluded from further studies. The potential coregulation of *TkSQS1* and *TkSQE1* expression in *T. koksaghyz* latex was investigated by growing wild‐type plants in the greenhouse and isolating latex from 8‐ to 20‐week‐old plants. RNA was pooled from three individual plants and three pools were analyzed by qRT‐PCR. *TkSQS1* and *TkSQE1* shared near‐identical expression profiles with expression peaking in 12‐ and 16‐week‐old plants (Figure 3c). In addition to the accumulation of triterpenoids during this growth period (Figure 3d,f) we also analyzed the expression of the OSC gene *TkOSC1* (GenBank: MG646376), which was recently shown to be responsible for the synthesis of the major pentacyclic triterpenes in dandelion latex. Although the squalene content increased in 10‐ and 12‐week‐old wild‐type *T. koksaghyz* plants, 2,3‐oxidosqualene continued to accumulate until the plants were 16 weeks old and then declined (Figure 3d). Moreover, although the sterol content remained nearly constant, the quantity of pentacyclic triterpenes continued to decline with plant age (Figure 4f). Interestingly, the pentacyclic triterpenes which decreased most were α‐amyrin, β‐amyrin and taraxasterol (Supporting Information Table S6). Accordingly, *TkOSC1* expression was highest in 10‐ and 12‐week‐old *T. koksaghyz* plants and declined thereafter (Figure 3e). In addition, we also investigated whether treatment with methyl jasmonate (MeJA) was able to induce *TkSQS1*–*TkSQE2* and *TkSQE1*–*TkSQE4* gene expression in 8‐week‐old *T. koksaghyz* wild‐type plants (Supporting Information Table S4). Here, *TkSQS2* and *TkSQE2* showed no expression in *T. koksaghyz* latex, whereas *TkSQE3*–*TkSQE4* were expressed at low levels in MeJA‐treated plants, but displayed no significant difference in gene expression compared to control plants. *TkSQS1* and *TkSQE1* gene expression was slightly elevated in MeJA‐treated plants compared to the control plants, but again not significantly.

### 3.3 Localization and enzymatic activity of TkSQS1 and TkSQE1

Next we investigated the subcellular localization of TkSQS1 and TkSQE1 by transient expression in *N. benthamiana* epidermal leaf cells followed by the functional analysis of enzyme activity using in vitro and yeast complementation assays for TkSQS1 and TkSQE1, respectively.

CLSM analysis revealed that the Cerulean‐TkSQS1 fusion protein colocalized with NtermCYP51G1‐mRFP, but not with the cytosolic mRFP marker (Figure 4a–b and Supporting Information Figure S3a–b). This is indicated by merged images and quantitative analysis of the fluorescent signals in regions of interest. As a negative control, cytosolic Cerulean was coinfiltrated with NtermCYP51G1‐mRFP to ensure that the ER localization of TkSQS1 was not elicited by the fusion with Cerulean (Supporting Information Figure S3e). Likewise, the C‐terminal TkSQE1 fusion protein (TkSQE1‐Cerulean) colocalized with NtermCYP51G1‐mRFP, but not with cytosolic mRFP (Figure 4c–d and Supporting Information Figure S3c–d).

The N‐terminal TkSQE1 fusion protein (Cerulean‐TkSQE1) aggregated in the tobacco cells, indicating that normal protein folding was disrupted by the fusion. Therefore, the potentially distinct role of the two TkSQE1 transmembrane domains in the localization of the enzyme could not be evaluated. However, our results clearly showed that both enzymes localize to the ER membrane in the heterologous *N. benthamiana* expression system.

**Figure 5:**
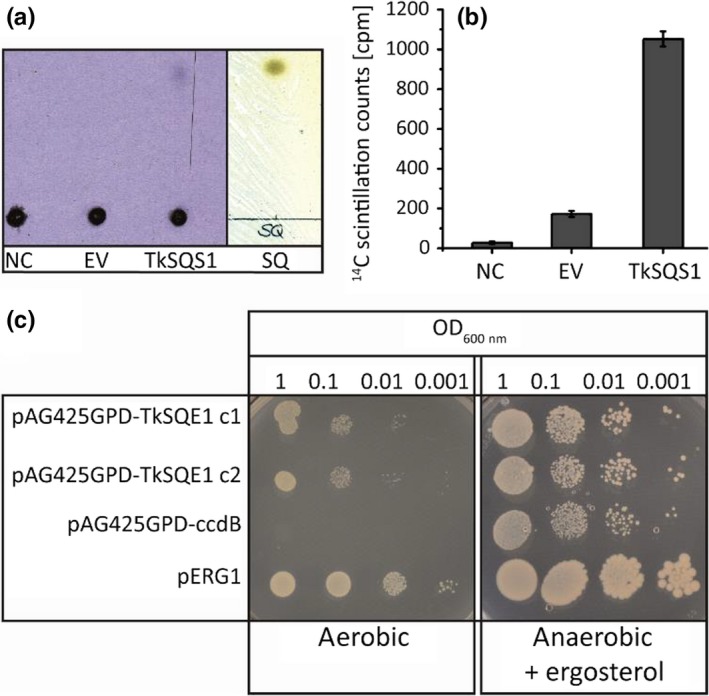
Functional analysis of TkSQS1 and TkSQE1. TkSQS1 (a–b) activity assay and TkSQE1 complementation (c). (a) Phosphor imaging with negative control (NC), empty vector control (EV) and TkSQS1 protein extracts and corresponding reversed‐phase TLC plate showing squalene standard visualized with iodine vapor. (b) Silica was scraped from the TLC plate at the height of the squalene standard, and radioactivity was measured by scintillation counting. (c) Complementation of the yeast strain KLN1 (Δ*erg1*) to confirm the activity of TkSQE1. The yeast was transformed with the vector pAG425GPD‐TkSQE1 or the negative control vector pAG425GPD‐ccdB. KLN1‐pERG1 complemented with the endogenous ERG1 served as a positive control. Two colonies of KLN1‐TkSQE1 and one colony of KLN1‐pERG1 and KLN1‐ccdB were selected, dropped out, and incubated for 3 days under anaerobic conditions in the presence of ergosterol, or incubated for 5 days under aerobic conditions without supplements

To confirm the functionality of TkSQS1, an in vitro SQS assay was carried out with [1,2‐^14^C]‐labeled FPP as the substrate in protein extracts from *N. benthamiana* leaves which was quantified by scintillation counting (Figure 5a). Proteins extracted from *TkSQS1*‐infiltrated *N. benthamiana* leaves showed a sevenfold increase in squalene production compared to the infiltrated empty vector control, which confirmed the activity of TkSQS1 (Figure 5b).

The squalene epoxidase activity of TkSQE1 was confirmed in a yeast complementation assay. *S. cerevisiae* strain KLN1 (Δ*erg1*) (Lipinski et al., 2009) was transformed with the vector pAG425GPD‐TkSQE1 or the empty vector pAG425GPD‐ccdB as a negative control, and KLN1‐pERG1 served as positive control. *ERG1* encodes the *S. cerevisiae* SQE, so that KLN1 (Δ*erg1*) is depleted for essential sterols and can only grow on ergosterol under anaerobic conditions. KLN1 complementation with TkSQE1 was feasible and resulted in growth under aerobic conditions, whereas the negative control was only able to grow under anaerobic conditions with ergosterol provided as a supplement (Figure 5c). These data confirmed the functionality of TkSQE1.

### 3.4 *TkOSC1* expression is downregulated in TkSQS1‐RNAi and TkSQE1‐RNAi plants

Having identified the *TkSQS1* and *TkSQE1* genes and functionally characterized the corresponding enzymes, we investigated the effect of silencing the expression of either *TkSQS1* or *TkSQE1* in *T. koksaghyz* plants by RNAi.

**Figure 6:**
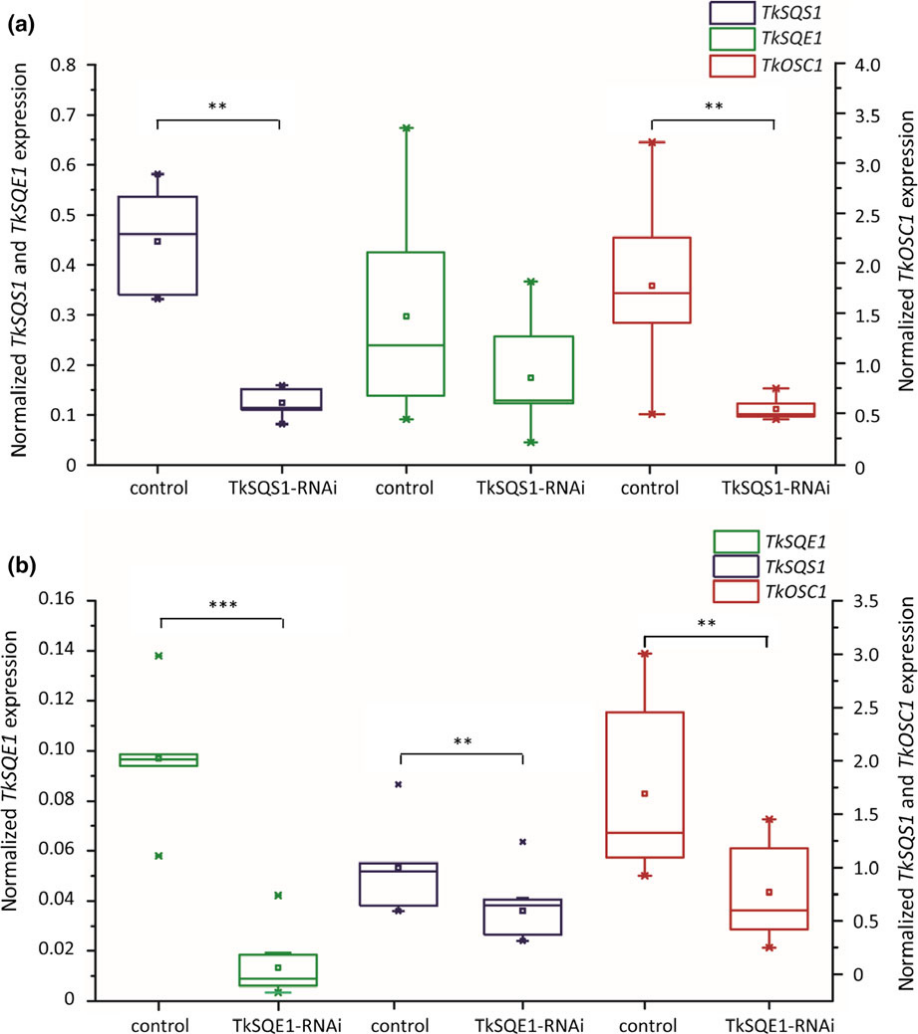
*TkSQS1*,* TkSQE1,* and *TkOSC1* mRNA expression analysis in latex from TkSQS1‐RNAi and TkSQE1‐RNAi *Taraxacum koksaghyz* plants. The corresponding mRNA levels were normalized against the constitutive genes encoding elongation factor 1α (*TkEF1*α) and ribosomal protein L27 (*TkRP*) from *T. koksaghyz*. The transgenic plants were analyzed 12 weeks after sowing. *TkSQS1*,* TkSQE1,* and *TkOSC1* mRNA expression analysis in (a) TkSQS1‐RNAi plants (*n* = 10 from six independent lines) compared to control plants (*n* = 7) and (b) TkSQE1‐RNAi plants (*n* = 11 from four independent lines) compared to control plants (*n* = 6). Asterisks denote statistical significance compared to control (two‐tailed *t* test, ** = *p* < 0.01, *** = *p* < 0.001)

We characterized 13 TkSQS1‐RNAi and 4 TkSQE1‐RNAi transgenic lines in terms of T‐DNA insertion and gene expression. Transgenic T0 plants were pollinated using a wild‐type plant for seed generation, and 10 plants from six different TkSQS1‐RNAi lines with a strong silencing effect were selected and analyzed in the T1 generation. *TkSQS1* expression in the latex harvested from roots was measured by qRT‐PCR in 12‐week‐old plants because this time point showed the highest expression level in wild‐type plants (Figure 3c). Another seven TkSQS1‐RNAi lines displaying no RNAi effect were used as negative controls. The 10 lines showing a strong RNAi effect and the seven controls were used for the further analysis of gene expression and metabolite levels. Putative regulatory effects at the transcriptional level were determined by analyzing *TkSQS2*,* TkSQE1,* and *TkOSC1* gene expression (Figure 6a). *TkSQS2* expression was not detected in latex samples from 12‐week‐old wild‐type plants, indicating that *TkSQS2* does not compensate for the reduction of *TkSQS1* expression. Therefore, TkSQS1 and TkSQS2 do not appear to be functionally redundant.

*TkSQE1*, as the major *SQE* gene expressed in the latex, showed a slight but not statistically significant reduction in expression in the TkSQS1‐RNAi lines compared to the control plants. Remarkably, we also observed a positive correlation between *TkOSC1* and *TkSQS1* expression in the TkSQS1‐RNAi lines (Figure 6a). Moreover, gene expression in the root material of TkSQS1‐RNAi lines was assessed for *TkSQS1*‐*2*,* TkSQE1*,* TkSQE3,* and *TkOSC1* expression (Supporting Information Figure S4a). In all transgenic lines, the expression of *TkSQS1*,* TkSQE1*,* TkSQE3,* and *TkOSC1* was similar or lower compared to control plants, while *TkSQS2* showed no expression at all so that compensatory gene induction in root tissue can be excluded.

For the analysis of TkSQE1‐RNAi lines, we selected a set of 11 T2 transgenic plants representing four independent transgenic lines. Because all the transgenic plants showed an RNAi effect, six nontransgenic plants from the same generation were used as controls. Subsequent qPCR analysis revealed a significant reduction in *TkSQE1* expression to 20% of the level in wild‐type plants (Figure 6b). At the same time, the expression levels of *TkSQS1* and *TkOSC1* were significantly reduced, which indicated the coregulation of *TkSQS1*,* TkSQE1,* and *TkOSC1* as suggested by the analysis of the TkSQS1‐RNAi lines. The expression of *TkSQE2–TkSQE4* was also examined, and there were no differences from the control plants, indicating that *TkSQE2–TkSQE4* do not compensate for the reduction in *TkSQE1* expression. In addition, gene expression in root material of TkSQE1‐RNAi lines was analyzed for *TkSQS1*‐*2*,* TkSQE1*,* TkSQE3,* and *TkOSC1* expression (Supporting Information Figure S4b). In all transgenic lines, the expression of *TkSQS1*,* TkSQE3,* and *TkOSC1* was lower compared to control plants although not significantly, whereas *TkSQE1* displayed a significantly lower expression level in transgenic compared to wild‐type plants. Moreover, *TkSQS2* could not be detected in either transgenic or in wild‐type root material.

**Figure 7:**
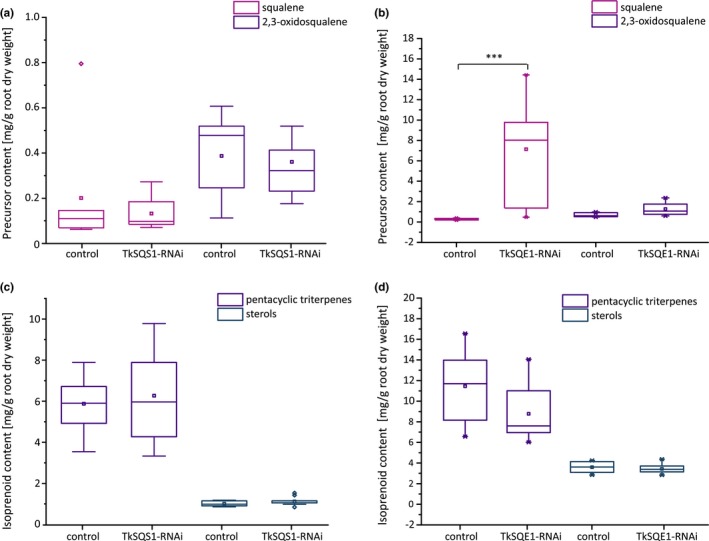
Metabolic analysis of TkSQS1‐RNAi and TkSQE1‐RNAi plants. Squalene and 2,3‐oxidosqualene levels in freeze‐dried root material were determined by GC‐MS in (a) TkSQS1‐RNAi (*n* = 10 from six independent lines) and corresponding control plants (*n* = 7) and in (b) TkSQE1‐RNAi (*n* = 11 from four independent lines) and corresponding control plants (*n* = 6). Pentacyclic triterpene and sterol levels in freeze‐dried root material determined by GC‐MS in (c) TkSQS1‐RNAi (*n* = 10 from six independent lines) and corresponding control plants (*n* = 7) and in (d) TkSQE1‐RNAi (*n* = 11 from four independent lines) and corresponding control plants (*n* = 6). Pentacyclic triterpenes = taraxerol, α‐amyrin, β‐amyrin, lupeol and taraxasterol; sterols = campesterol, stigmasterol and γ‐sitosterol. Asterisks denote statistical significance compared to control (two‐tailed *t* test, ** = *p* < 0.01, *** = *p* < 0.001)

In addition, roots of 12‐week‐old TkSQS1‐RNAi and TkSQE1‐RNAi plants were cut directly below the rosette, freeze‐dried, ground, and used for the extraction of metabolites for analysis by gas chromatography‐mass spectrometry (GC‐MS). Interestingly, the squalene and 2,3‐oxidosqualene levels in 12‐week‐old TkSQS1‐RNAi transgenic lines were not significantly different from the levels in the control lines (Figure 7A, Supporting Information Table S7). The same was true for the levels of pentacyclic triterpenes and sterols (Figure 7c, Supporting Information Table S7). However, GC‐MS analysis of TkSQE1‐RNAi roots revealed a 27‐fold increase in squalene levels compared to control plants, but no significant differences in the levels of 2,3‐oxidosqualene (Figure 7b, Supporting Information Table S8).

The *TkSQE1* expression profile and the accumulation of squalene confirmed the effectiveness of this RNAi approach. Surprisingly, neither the sterol content nor the amount of pentacyclic triterpenes was significantly reduced (Figure 7d, Supporting Information Table S8). Like the TkSQS1‐RNAi lines, the TkSQE1‐RNAi lines did not show significant differences in the accumulation of isoprenoid end‐products compared to controls, although *TkSQE1* expression and the accumulation of squalene were knocked down successfully.

## 4 DISCUSSION

We have carried out a detailed analysis of the *SQS* and *SQE* genes in *T. koksaghyz* and a functional characterization of their products. In silico analysis of the *TkSQS1*,* TkSQS2,* and *TkSQE1–TkSQE4* cDNA sequences revealed high similarities to other known SQS and SQE sequences and the presence of known catalytically active domains indicating functional enzyme activity (Supporting Information Figure S1). Interestingly, TkSQS1 and TkSQS2 did not cluster together in the same branch of the SQS phylogenetic tree, suggesting that gene duplication may have predated speciation, hinting at a specialized tissue‐specific role or sub‐functionalization (Figure 2). Similar results have been obtained for SQS proteins from *G. glabra* in phylogenetic analyses (Hayashi et al., 1999; Navarro Gallón et al., 2017). However, this contrasts with the *M. domestica* MdSQS1 and MdSQS2 isoforms, which show higher intraspecific identities and may indicate more recent gene duplication events (Navarro Gallón et al., 2017).

Similarly, TkSQE3 and TkSQE4 did not cluster closely with other plant SQEs (Figure 2). This could indicate a specialized function which should be assessed in future studies, as already proposed for *A. thaliana SQE* genes (Laranjeira et al., 2015; Rasbery et al., 2007). Tissue‐specific expression analysis revealed that *TkSQS1* and *TkSQE1* are the predominant latex isoforms (Figure 3a,b) and are therefore likely to play a key role in dandelion latex isoprenoid biosynthesis. The multitissue expression of *SQS* and *SQE* is common and has been described for *SQS* and *SQE* genes in *A. thaliana* (Busquets et al., 2008; Rasbery et al., 2007), *P. ginseng* (Han et al., 2010; Lee et al., 2004) and *W. somnifera* (Bhat et al., 2012; Razdan et al., 2013) among others, mirroring our results in *T. koksaghyz*.

Infiltration studies confirmed the localization of TkSQS1 and TkSQE1 on the ER membrane (Figure 4) as postulated for other SQS and SQE enzymes (Busquets et al., 2008; Kajikawa et al., 2015; Laranjeira et al., 2015; Leber et al., 1998). The in vitro TkSQS1 activity assay and the complementation of the impaired yeast strain KLN1 (Δ*erg1*) by TkSQE1 confirmed the activities of both *T. koksaghyz* enzymes (Figure 5).

Comparative expression analysis revealed that *TkSQS1* and *TkSQE1* share similar latex‐preferred expression profiles during dandelion development (Figure 3c). Such coregulation may ensure that isoprenoid precursors are efficiently shuttled toward sterol or pentacyclic triterpene biosynthesis. In addition, minor effects of higher *TkSQS1* and *TkSQE1* gene expression in MeJA‐treated plants compared to control plants could hint at an involvement in defense against herbivores or pathogens as has been reported for other *T. koksaghyz* genes involved in secondary metabolite biosynthesis (Cao et al., 2017). Moreover, expression peaks in 12‐ and 16‐week‐old plants indicated an increase in the demand for sterols and/or pentacyclic triterpenes, perhaps due to the rapid expansion of root volume (Schaller, 2003) or the rapid growth of laticiferous cells, in turn requiring the efficient shuttling of precursors via coordinated expression. Similar studies in *T. brevicorniculatum* revealed that genes in the mevalonate pathway also show similar expression patterns and expression peaks in 16‐week‐old plants (Pütter et al., 2017). Thus, membrane sterols seem to be required at this stage of development and are provided by the mevalonate pathway and subsequent SQS, SQE and OSC activity. Additional post‐transcriptional mechanisms also seem to play a role in SQE activity. Although the squalene content during *T. koksaghyz* development increases in 10‐ to 12‐week‐old plants and then declines to a low level, 2,3‐oxidosqualene levels increase until the plants are 16 weeks old before declining (Figure 3d). Therefore, although *TkSQS1* and *TkSQE1* expression levels remain similar at these times, further stabilization mechanisms or post‐translational modifications may be required to increase the total 2,3‐oxidosqualene content of the roots.

To gain insight into the role of TkSQS1 and TkSQE1 in dandelion isoprenoid biosynthesis, we generated RNAi lines in which these genes were silenced (Figures 6 and 7). TkSQS1‐RNAi lines showed significantly lower *TkSQS1* expression, and surprisingly the expression levels of the genes *TkSQE1* and *TkOSC1* were simultaneously reduced, although the downregulation of *TkSQE1* was not statistically significant. A similar correlation was observed for the TkSQE1‐RNAi lines, in which the expression levels of *TkSQS1*,* TkSQE1,* and *TkOSC1* were significantly reduced compared to control lines. These findings indicate that *TkSQS1*,* TkSQE1,* and *TkOSC1* are coregulated in *T. koksaghyz* latex, as reflected by the similar developmental expression profiles of *TkSQS1* and *TkSQE1* (Figure 3c). Transcriptional coregulation may increase the flux toward isoprenoid biosynthesis and is one of several mechanisms that can be used to regulate the production of isoprenoids.

In *P. ginseng*, the overexpression of *SQS* resulted in higher expression levels of *SQE*, β*AS* (*OSC*) and cycloartenol synthase, which also support the positive coregulation of genes in the isoprenoid biosynthesis pathway (Lee et al., 2004). In addition, the virus‐induced gene silencing of *W. somnifera SQS* reduced the expression of several downstream genes including *WsSQE*, whereas upstream genes were induced. Contrary to our results, the expression of *Ws*β*AS1* was upregulated whereas cycloartenol synthase was downregulated (Singh et al., 2015). Therefore, although the regulation of OSCs may differ in a species‐dependent manner, there appears to be a positive correlation between OSC and SQS/SQE expression in *T. koksaghyz* latex.

To investigate the regulation of isoprenoid biosynthesis in more detail, freeze‐dried root material from the RNAi lines was evaluated to determine the content of triterpene precursors, sterols and pentacyclic triterpenes (Figure 7). The quantity of pentacyclic triterpenes was not affected by the silencing of *TkSQS1* or *TkSQE1*, perhaps reflecting the overall high‐level accumulation of pentacyclic triterpenes in the early stages of dandelion development. The quantity of pentacyclic triterpenes peaked at 26.8 mg/g dry weight (dw) in 8‐week‐old *T. koksaghyz* wild‐type plants before declining with age (Figure 3f), but high levels were still present after 12 weeks (17.2 mg/g dw). *TkSQS1* and *TkSQE1* silencing and the reduced expression of *TkOSC1* therefore do not have a profound effect on total pentacyclic triterpene levels in 12‐week‐old plants. In contrast, the remarkable impact of *TkSQE* silencing on squalene accumulation was clearly seen at this developmental stage, given that squalene is present at only low levels in 12‐week‐old wild‐type plants (0.2 mg/g dw). This effect was previously reported in *A. thaliana Atsqe1* mutants (Rasbery et al., 2007) and demonstrates the reduced flux between squalene and 2,3‐oxidosqualene elicited by TkSQE1. Squalene accumulation as a successful indication of SQE inhibition or knockdown has also been observed in other species: the treatment of tobacco BY‐2 cells with terbinafine strongly increased squalene levels, as did artificial microRNA‐mediated *SQE* silencing and terbinafine treatment in *Chlamydomonas reinhardtii* (Kajikawa et al., 2015; Wentzinger, Bach, & Hartmann, 2002). Interestingly, although the inhibition of SQE reduced sterol levels, the gene silencing approach failed to reduce sterol levels significantly, which was attributed to residual SQE activity in the *C. reinhardtii* knockdown lines (Kajikawa et al., 2015). Sufficient SQE activity in *T. koksaghyz* latex might also explain the unaltered isoprenoid end‐product levels, possibly due to post‐translational regulation. The expression of *TkSQS2* and *TkSQE2–4* did not change in the RNAi lines, indicating that other *SQS* and *SQE* genes are not upregulated in latex to compensate for the depletion of *TkSQS1* and *TkSQE1* (Han et al., 2010). Moreover, *SQS* and *SQE* gene expression in the root tissue of the RNAi lines also did not compensate for the corresponding reduced latex‐predominant expression of *TkSQS1* and *TkSQE1*, so that compensatory expression in adjacent tissues can be excluded (Supporting Information Figure S4).

However, because the conversion of squalene to 2,3‐oxidosqualene occurs at a constant rate, post‐translational regulation of SQE might increase the overall stability of the enzyme to ensure that the levels of downstream isoprenoids remain constant.

Human SQE is regulated by cholesterol‐dependent proteasomal N‐terminal degradation on the ER membrane (Chua, Howe, Jatana, Thukral, & Brown, 2017; Gill, Stevenson, Kristiana, & Brown, 2011). In yeast, a similar feedback mechanism is known to be lanosterol‐dependent (Foresti, Ruggiano, Hannibal‐Bach, Ejsing, & Carvalho, 2013). A related mechanism may exist in plants to promote the degradation of SQEs when isoprenoid end‐products such as sterols are present. When these end‐products are depleted, the enzyme may be stabilized until a certain quantity of end‐products accumulates again. Despite low *SQE* expression levels, this could stabilize the enzyme and would explain the lack of an end‐product effect in our RNAi lines. The activation or stabilization of TkSQS1 and TkSQE1 through such a post‐translational feedback mechanism at the ER membrane will be investigated in the future.

## 5 CONCLUSIONS

We analyzed the *T. koksaghyz* enzymes SQS and SQE. The latex‐preferred isoforms TkSQS1 and TkSQE1 were knocked down by RNAi but this was not sufficient to cause an effect on isoprenoid biosynthesis even though the *TkSQS1*,* TkSQE1* and *TkOSC1* genes were downregulated in both RNAi approaches. These findings indicate that pentacyclic triterpene biosynthesis is facilitated by efficient coregulation at the level of transcription.

## CONFLICT OF INTEREST

The authors declare that they have no competing interests.

## AUTHORS’ CONTRIBUTIONS

N.v.D. and C.S.G. conceived and designed the experiments. K.U., K.M.P., and K.V. performed the experiments. C.S.G. and N.v.D. analyzed the data. K.U., K.M.P., N.v.D., and R.M.T. wrote the manuscript. D.P. and C.S.G supervised and complemented the writing.

## ACCESSION NUMBERS

TkSQS1 (MG646369), TkSQS2 (MG646370), TkSQE1 (MG646371), TkSQE2 (MG646372), TkSQE3 (MG646373), TkSQE4 (MG646374), TkOSC1 (MG646376).
