# Supplementary material for: Functional characterization of squalene synthase and squalene epoxidase in Taraxacum koksaghyz
Source: Plant Direct. 2018 Jun 13;2(6):e00063. doi: 10.1002/pld3.63 (PMC6508512; doi:10.1002/pld3.63)
Supplement: Supplementary file 1 [file PLD3-2-e00063-s001.docx]

**A**

ZmSQS1 MGALS----RPEEVLALVKLRVAAGQIKRQIPPEEHWAFAYSMLQKVSRSFALVIQQLGP 56

TkSQS2 MGTLKAVLQHPDDFYPLLKLMMAVKQAEKQIPALPHWGFCYSMLHKVSRSFGLVIQQLGS 60

AtSQS1 MGSLGTMLRYPDDIYPLLKMKRAIEKAEKQIPPEPHWGFCYSMLHKVSRSFSLVIQQLNT 60

AaSQS MSSLKAVLKHPDDFYPLLKLKMAAKKAEKQIPSQPHWAFSYSMLHKVSRSFALVIQQLNP 60

TkSQS1 MGSLKAVLKHPDDFFPLLKLKIAAKKAEKQIPAEPHWGFCYSMLHKVSRSFALVIQQLNP 60

HaSQS MGSLKAVLKHPDDFYPLLKLKMAAKKAEKQIPAEPHWGFCYSMLHKVSRSFALVIQQLNP 60

NtSQS MGSLRAILKNPDDLYPLVKLKLAARHAEKQIPPSPHWGFCYSMLHKVSRSFALVIQQLPV 60

HbSQS MGSLGAILKHPDDFYPLLKLKMAVRHAEKQIPPEPHWGFCYSMLHKVSRSFALVIQQLGP 60

*.:* *::. .*:*: * : :.***. **.*.****:******.******

ZmSQS1 ELRNAVCIFYLVLRAL**D**TVE**D**DTSIPTEVKVPILQEFYRHIYNRDWHYSCGTNHYKMLMD 116

TkSQS2 ELRDAVCVFYLLLRAL**D**TVE**D**DTTIDTMTKIPILMEFHRHIYDPYWHFSCGTKEYKILMD 120

AtSQS1 ELRNAVCVFYLVLRAL**D**TVE**D**DTSIPTDEKVPILIAFHRHIYDTDWHYSCGTKEYKILMD 120

AaSQS QLRDAVCIFYLVLRAL**D**TVE**D**DTSIAADIKVPILIAFHKHIYNRDWHFACGTKEYKVLMD 120

TkSQS1 ELRDAVCIFYLVLRAL**D**TVE**D**DTSIEADIKVPILIAFHEHIYDRDWHFACGTKEYKVLMD 120

HaSQS ELRDAVCIFYLVLRAL**D**TVE**D**DTSIDADIKVPILIAFHQHIYDRDWHFACGTKEYKVLMD 120

NtSQS ELRDAVCIFYLVLRAL**D**TVE**D**DTSIPTDVKVPILISFHQHVYDREWHFSCGTKEYKVLMD 120

HbSQS QLRNAVCIFYLVLRAL**D**TVE**D**DTSIPTDVKVPILIAFHRHIYDCEWHFSCGTKDYKVLMD 120

:**:***:***:***********:* : *:*** *: *:*: **::***: **:***

ZmSQS1 KFRHVSTAFLELGEGYQKAIEEVTRRMGAGMAKFICK-EVETVDDYDEYCH**Y**VAGLVGYG 175

TkSQS2 RFHHVSTAFLELNASFQEAIEDITMKMGAGMAKFICKEEVVSIADYDEYCH**Y**VAGHVGLG 180

AtSQS1 QFHHVSAAFLELEKGYQEAIEEITRRMGAGMAKFICQ-EVETVDDYDEYCH**Y**VAGLVGLG 179

AaSQS QFHHVSTAFLELKRGYQEAIEDITMRMGAGMAKFICK-EVETVDDYDEYCH**Y**VAGLVGIG 179

TkSQS1 QFHHVSAAFLELKKSYQEAIKDITMRMGAGMAKFICK-EVETVDDYDEYCH**Y**VAGLVGLG 179

HaSQS QFHHVATAFLELKKGYQEAIEDITKRMGAGMAKFICK-EVETVDDYDEYCH**Y**VAGLVGLG 179

NtSQS QFHHVSTAFLELRKHYQQAIEDITMRMGAGMAKFICK-EVETTDDYDEYCH**Y**VAGLVGLG 179

HbSQS QFHHVSTAFLELGKSYQEAIEDITKRMGAGMAKFICK-EVETVDDYDEYCH**Y**VAGLVGLG 179

.*.**::***** :*:**:::* .**********: ** : *********** ** *

ZmSQS1 LSRLFYAAGTEDLALD--SLSNSMGLFLQKTNIIR**D**YLE**D**INEIPKSRMFWPREIWSKYA 233

TkSQS2 LSKLFHASGKEVLFPE--STSNAMGLFLQKTNIVR**D**FLV**D**INEVPKSRVYWPRQVWSRYA 238

AtSQS1 LSKLFLAAGSEVLTPDWEAISNSMGLFLQKTNIIR**D**YLE**D**INEIPKSRMFWPREIWGKYA 239

AaSQS LSKLFHSSGTEILFSD--SISNSMGLFLQKTNIIR**D**YLE**D**INEIPKSRMFWPREIWSKYV 237

TkSQS1 LSKLFHASGTEKLFPD--SISNSMGLFLQKINIIR**D**YLE**D**INEIPKSRMFWPREIWSKYV 237

HaSQS LSKLFHASGTEILFPD--SMSNSMGLFLQKTNIIR**D**YLE**D**INEIPKSRMFWPREIWSKYV 237

NtSQS LSKLFHASGKEDLASD--SLSNSMGLFLQKTNIIR**D**YLE**D**INEVPKCRMFWPREIWSKYV 237

HbSQS LSKLFHASGSEDLAPD--VLSNSMGLFLQKTNIIR**D**YLE**D**INEIPKSRMFWPRQTWSKYV 237

**.** ::*.* * : **:******* **:**:* ****:**.*::***: *..*.

ZmSQS1 DKLEDFKYEENSKKAVQCLNNMVTDALIHAEECLQYMSALKD**P**AI**F**R**F**CAIP**Q**IMAIGTC 293

TkSQS2 SKPEDLKHKDNSKKAVQCLNHMVTNALVHIEDCLKYLSGIRD**P**AI**F**K**F**CAIP**Q**IMAIGTL 298

AtSQS1 DKLEDLKYEENTNKSVQCLNEMVTNALMHIEDCLKYMVSLRD**P**SI**F**R**F**CAIP**Q**IMAIGTL 299

AaSQS NKLEDLKYEENSEKAVQCLNDMVTNALIHIEDCLKYMSQLKD**P**AI**F**R**F**CAIP**Q**IMAIGTL 297

TkSQS1 NKLEELKYEENSDKAVQCLNDMVTNALIHIEDCLKYMSELRD**P**AI**F**R**F**CAIP**Q**IMAIGSL 297

HaSQS NKLEDFKYEDNSEKAVQCLNDMVTNALIHIEDCLKYMSDLRD**P**AI**F**R**F**CAIP**Q**IMAIGTL 297

NtSQS NKLEELKYEDNSAKAVQCLNDMVTNALSHVEDCLTYMSALRD**P**SI**F**R**F**CAIP**Q**VMAIGTL 297

HbSQS NKLEDLKDEENSVKAVQCLNDMITNALIHVDDCLTYMSALRD**P**AI**F**R**F**CAIP**Q**VMAIGTL 297

.* *::* ::*: *:***** *:*:** * ::** *: :.**:**.******:****:

ZmSQS1 ALCYNNVHVFRGVVKMRRGLTARIIDETNSMTDVYTAFYEFSSLIESKIDDSDPNAALTQ 353

TkSQS2 ALCYNNIEVFRGVVKLRRGLTAKIIDRTNTMADVYGAFNDFCSMIRSKINVKDPNAHMTV 358

AtSQS1 ALCYNNEQVFRGVVKLRRGLTAKVIDRTKTMADVYGAFYDFSCMLKTKVDKNDPNASKTL 359

AaSQS ALCYNNIEVFRGVVKLRRGLTAKVIDRTKTMADVYQAFSDFSDMLKSKVDMHDPNAQTTI 357

TkSQS1 ALCYNNIEVFRGVVKMRRGLTAKVIDRTKTMADVYRAFYDFSSMLKAKVDMNDPNAKTTI 357

HaSQS ALCYNNVQVFQGVVKMRRGLTAKVIDRTRTMGDVYQAFYDFSSMLKSKVDMRDPNATTTI 357

NtSQS AMCYDNIEVFRGVVKMRRGLTAKVIDQTRTIADVYGAFFDFSCMLKSKVNNNDPNATKTL 357

HbSQS ALCYNNIEVFRGVVKMRRGLTAKVIDQTKTMADVYGAFFDFSCMLKSKVDRSDPNAEKTF 357

*:**:* **.****:******.:** *.:: *** ** :*. :: :*:: **** *

ZmSQS1 RRVDSIKQTCKSSG-LVKQRGYHLEKSP-YRPMLIMIVLLLVAILFGVMF----------- 401

TkSQS2 SRIDDIQKICKNSRTLDKWKSYIIDDKPTCRPALIAFLIVIMAILYAYSQHHKSKEATS-- 417

AtSQS1 NRLEAVQKLCRDAGVLQNRKSY-VNDKGQPNSVFIIMVVILLAIVFAYLRAN--------- 410

AaSQS TRLEAAQKICKDSGTLSNRKSYIVKRESSYSAALLALLFTILAILYAYLSANRPNKIKFTL 418

TkSQS1 TRIEAAQRICKDSGTLTNRRSYIVKSEPSYSPALIALVFIILAILYAYMSANRTNKFKVTL 418

HaSQS TRIEAAQKICKDSGALNNRKSYIVESEPSYSPALIALLFIILAILYAYTSANRQNKIKVTL 418

NtSQS KRLEAILKTCRDSGTLNKRKSYIIRSEPNYSPVLIVVIFIILAIILAQLSGNRS------- 411

HbSQS SRVEAIQKTCRESGLLNKRKSYIIRNKPRYNPALIILLVIILSIIFAYRSGNQASN----- 413

*:: . *..: * :...* : . . :: .:. :::*: .

**B**

TkSQE4 ------------------------------------------------------------ 0

TkSQE3 MVPIKPATYSTISLGFTGYRSHATHYTHKTHNRILSNTCHRSINRRGQEPPPLISSSSVP 60

AtSQE1 ------------------------------------------------------------ 0

TkSQE2 ------------------------------------------------------------ 0

TkSQE1 ------------------------------------------------------------ 0

HaSQE ------------------------------------------------------------ 0

PgSQE1 ------------------------------------------------------------ 0

PgSQE2 ------------------------------------------------------------ 0

TkSQE4 --------MELK-------ILQQYILGGIIATWLGFTLIYLL--GVKKSKPDDGLHV--- 40

TkSQE3 RLTSSTKVLTSKRKMMENTIDNSYIVATFFVSFLGFVVLS-----VLRRRTSYLISYS-- 113

AtSQE1 --------MESQ-------LWNWILPLLISSLLISFVAFYGF---FVKPKRNGLRH---- 38

TkSQE2 -------------------MADRLMLASIFVSVVGLILFWIS---ILIKNGRP------- 31

TkSQE1 --------MELGLPLVI--HDNYHLFVSAVALLLGFVLLYGLRSNLKTRRKSKEIW---- 46

HaSQE --------MELESPTTVI-HQNYLLLASAVALLTGFLLLYIL---ILNSRKSTEIH---- 44

PgSQE1 MNSSSSSTTDTLHSFMEALLIDQYFLGWIFAFLFGFLLLLNF---KRKREKNN------- 50

PgSQE2 --------MELERSYR---ENDEYFLMFAATLLFGFVLYLFT---LRRRRRRREKKGGAG 46

: : .:

TkSQE4 --------HKQTNELVRT---QPQKDGGI----------DVIIV**G**A**G**VA**G**AALAYSLGKD 79

TkSQE3 --------KKYVSGNNNSSKNRSNLIRGEFRQRYGSVDDDVIIV**G**A**G**VA**G**AALAHTLGKQ 165

AtSQE1 --------DRKTVSTVTSDVGSVNITGDT--------VADVIVV**G**A**G**VA**G**SALAYTLGKD 82

TkSQE2 --------HKSASVTTKSTSISTECRSGD--------ETDVIIV**G**A**G**VA**G**AALAHTLAKD 75

TkSQE1 --------RKSSNNSGNDGLQLPETDGST----------DVIIV**G**A**G**VA**G**AALACTLAKD 88

HaSQE --------RKCVDNSGDDGLRLTET--------------DVIIV**G**A**G**VA**G**AALACTLAKD 82

PgSQE1 --------STEFGTDDSNGYYTPENIAGS---------TDVIIV**G**A**G**VA**G**SALAYTLAKD 93

PgSQE2 SMEIINGAYKMTSSSEVNGHCTPEDIAGS--------SDDVIIV**G**A**G**VA**G**SALAYTLAKD 98

: ***:*******:*** :*.*:

TkSQE4 GRRILVIERDLGLQDRIVGELLQPGGFMKLIDLGLEDCVEDIDAQKVFGYALFKDDKSTS 139

TkSQE3 GRKVRVIERDLTEPDRIVGELLQPGGYLKLIELGLEDCVEEIDAQRVLGYALFKDGKSTK 225

AtSQE1 KRRVHVIERDLSEPDRIVGELLQPGGYLKLLELGIEDCVEEIDAQRVYGYALFKNGKRIR 142

TkSQE2 GRRVLVIERDLTEPDRIVGELLQPGGYLKLMELGLEDCVEDIDAQRVFGYALFKDGKNTR 135

TkSQE1 GRRVHVIERDLTEPDRIVGELLQPGGYLKLIELGLQDCVDGIEAQQVFGYAIYMDGKNTK 148

HaSQE GRRVHVIERDLTEPDRIVGELLQPGGYLKLIELGLEDCVDGIEAQQVFGYAIYMDGRNTK 142

PgSQE1 GRRVHVIERDLTEQDRIVGELLQPGGYLKLIELGLEDCVNEIDAQRVFGYALYMDGKNTR 153

PgSQE2 GRRVHVIERDLTEQDRIVGELLQPGGYLKLVELGLEDCVNEIDAQRVFGYALYMDGKNTR 158

*.: ****** ************::**::**::***: *:**.* ***:: :..

TkSQE4 LAYPLEKYMRDVAGRSFHNGRFVQRMREKAATVSSVRLEEGTVTSLVEEKGVVRGVRYKT 199

TkSQE3 LSYPLQKFHSDVSGRSFHNGRFIQKMREKATTLPNVKLEQGTVTNLLEDEGTVRGVQFKT 285

AtSQE1 LAYPLEKFHEDVSGRSFHNGRFIQRMREKAASLPNVQLEQGTVLSLLEENGTIKGVRYKN 202

TkSQE2 LSYPLEEFHADVAGRSFHNGRFIQKMREKASSLPTIRLQQGTVVSLLEEEGTIKGALYKT 195

TkSQE1 LSYPLEKFTSDISGRSFHNGRFIQQMREKAKTLPNLKMEQGTVTSLFEKDGIVNGVCYKT 208

HaSQE LSYPLEKFESDISGRSFHNGRFIRRMREKAASLPNVKLEQGTVTSLLEEHGTVKGVCYKT 202

PgSQE1 LSYPLEKFHSDVAGRSFHNGRFVQRMREKAASLPNVRMEQGTVTSLVEKKASVKGVQYKT 213

PgSQE2 LSYPLEKFHADVAGRSFHNGRFIQRMREKAASLPNVRMEQGTVTSLVEQKGTVKGVRYKT 218

*:***::: *::*********:..***** ::..:.:::*** .*.*. . :.*. :*.

TkSQE4 KSGQDIT-ANAPLTIVCDGCFSNLRRGLCKPEVDIPSSFAALLLKNCEAPYPNHGHVILA 258

TkSQE3 KSGEIIN-AFAPLTIVCDGCFSNLRRSLCKPQVDVPSCFVGLILENCDLPYPDHGHVILA 344

AtSQE1 KAGEEQT-AFAALTIVCDGCFSNLRRSLCNPQVEVPSCFVGLVLENCNLPYANHGHVVLA 261

TkSQE2 KNGKEMK-AFAPLTIVCDGCFSNLRRSLCNPQVDVPSCFVGLILENCNLPYENHGHVVLA 254

TkSQE1 KDGQTLT-AHAPLTIVCDGCFSNLRRSLCKPKVEVPSCFVGLVLENIDLPYANHGHVILA 267

HaSQE KDGQVMT-AHAPLTIVCDGCFSNLRRALCKPKVEVPSCFVGLVLENVDLPYANHGHVILA 261

PgSQE1 KDGQELS-AFAPLTIVCDGCFSNLRRSLCNPKVEVPSCFVGLILENIDLPHVNHGHVILA 272

PgSQE2 KNGQEMSAAYAPLTIVCDGCFSNLRHSLCNPKVDVPSCFVGLILENIDLPHINHGHVILA 278

* *: . * *.*************..**:*:*::**.*..*:*:* : *: :****:**

TkSQE4 DPSPILCYRISSTEIRCLVDIPGKKIPSVGNGEMAIHLKTHVAPQIPYELRGGFIAAVDE 318

TkSQE3 NPSPILFYQISKTEVRCLVDIPGQNLPSVANGDMAKYLNSVVAPQVPVQVRNAFVAAIDK 404

AtSQE1 DPSPILMYPISSTEVRCLVDVPGQKVPSIANGEMKNYLKTVVAPQMPHEVYDSFIAAVDK 321

TkSQE2 DPSPILFYRISSTEIRCLVDVPGQKVPSISNGDLSKYLTTSVAPQIPTELKQSFISAVEK 314

TkSQE1 DPSPILFYPISNTEVRCLVDVPGQKVPSISNGEMATYLKTVVAPQIPPELYTAFVAAVDK 327

HaSQE DPSPILFYPISNTEVRCLVDVPGQKVPSIANGEMVDYLKTVVAPQVPPELYAAFVAAIDK 321

PgSQE1 DPSPILFYKISSTEIRCLVDVPGQKVPCISNGELANYLKTVVAPQVPKQLYNSFIAAVDK 332

PgSQE2 DPSPILFYKISSTEIRCLVDVPGQKVPSIANGELAHYLKTSVAPQIPPELYKSFIAAIDK 338

:***** * **.**:*****:**:::*.:.**:: :*.: ****:* :: .*::*:::

TkSQE4 GKIKTMANRSMPAAPQPTPGAILL**GD**SFNMRHPLTGGGMTVALSDVLIVRDLLRPLQKLN 378

TkSQE3 GNIRTMPNRTMPAVPVLPRGALLM**GD**AFNMRHPLTGGGMTVALSDIVELRDLLKPLRDFT 464

AtSQE1 GNIKSMPNRSMPASPYPTPGALLM**GD**AFNMRHPLTGGGMTVALADIVVLRNLLRPLRDLS 381

TkSQE2 GNIRTMPNRSMPAVPQPTPGALLM**GD**AFNMRHPLTGGGMTVALSDIVVLRDLLRPLRDLN 374

TkSQE1 GNIRTMPNRSMPADPQPTPGALLM**GD**AFNMRHPLTGGGMTVALSDIVLLRDLLRPLKNLN 387

HaSQE GNIRTMPNRSMPADPQPTPGALLM**GD**AFNMRHPLTGGGMTVALSDIVLLRDLLRPLSNLN 381

PgSQE1 GNIRTMPNRSMPADPHPTPGALLL**GD**AFNMRHPLTGGGMTVALSDIVLIRDLLRPLRDLH 392

PgSQE2 GKIKTMPNRSMPADPHSTPGALLL**GD**AFNMRHPLTGGGMTVALSDIVLIRDLLRPLRDLH 398

*:*.:*.**:*** * . **:*:**:****************:*:: :*:**.** .:

TkSQE4 DTIALCEYLNSFYTLRKPMASTINTLAGALYKVFSASPDEARTELRQACFDYLSLGGVFS 438

TkSQE3 DADSLSQNIESFYTLRKPVASTINTLAGALYRVFCASSDEAMKEMREACFGYLSLGGGCA 524

AtSQE1 DGASLCKYLESFYTLRKPVAATINTLANALYQVFCSSENEARNEMREACFDYLGLGGMCT 441

TkSQE2 DSYTLCKYLESFYTLRKPVASTINTLAGALYKVFCASPDKARQEMREACFDYLSLGGVFS 434

TkSQE1 DAPMLCHYLESFYTLRKPVSSTINTLAGALYKVFCASPDPARKEMRDACFDYLSLGGIYS 447

HaSQE DAPTLCNYLECFYTLRKPVSSTINTLAGALYKVFCASPDPARKEMRQACFDYLSLGGICS 441

PgSQE1 DSSTLCKYLESFYTLRKPVASTINTLAGALYKVFCASPDKARQEMRNACFDYLSLGGICS 452

PgSQE2 DSSTLCKYLESFYTLRKPVASTINTLAGALYKVFCASPDKARQEMRDACFDYLSLGGICS 458

* *. ::.*******:::******.***.**.:* : * *:*:***.**.*** :

TkSQE4 SGPIGLLSGLNPKPSSLVLHFFSVAVFGVGRLLLPFPSPQRTWLGVRLILDASGIIFPIL 498

TkSQE3 SGPVGLLSGLYPSPVQLVFHFFSVAVYGVFRLLLPLPSPNRLWIAARLILGAWGIIFPII 584

AtSQE1 SGPVSLLSGLNPRPLTLVCHFFAVAVYGVIRLLIPFPSPKRIWLGAKLISGASGIIFPII 501

TkSQE2 NGPVALLSGLNPRPISLVVHFFAVAVFGVGRLMLPFPSPKRIWIGARLISGASGIIFPII 494

TkSQE1 EGPISLLSGLNPRPLVLFLHFFAVAIYGVGRLLIPFPTPKRLWLGARLISGASGIIFPII 507

HaSQE QGPISLLSGLNPRPIVLFLHFFAVAIYGVGRLLIPFPSPKRLWLGARLISGASGIIFPII 501

PgSQE1 QGPIALLSGLNPRPISLFLHFFAVAIYGVGRLLIPFPSPKRMWLGARLILGASGIIFPII 512

PgSQE2 EGPIALLSGLNPRPMSLFFHFFAVAIYGVGRLLIPFPSPRKMWLGARLISGASGIIFPII 518

.**:.***** * * *. ***:**::** **::*:*:*.. *:...** .* ******:

TkSQE4 KAEGAAQMLLSSPVNPAYYKSPF------------ 521

TkSQE3 KDEGVRQMFFPATFPA-YYRGSSIVNFCLCVLFSL 618

AtSQE1 KAEGVRQMFFPATVPAYYYKAPTVGETKCS----- 531

TkSQE2 KAEGVRQMFFPATVAA-YYRAPPS----------- 517

TkSQE1 KSEGVRPMFFPATVPA-YYRSPRVA---------- 531

HaSQE KSEGVRQMFFPATVPA-YYKAPRVA---------- 525

PgSQE1 KSEGLRQMFFPATVPA-YYRAPPIH---------- 536

PgSQE2 KSEGVRQMFFPATVPA-YYRAPPITKKM------- 545

* ** *::.:.. . **...

**Figure S1: *In silico* analysis of TkSQS1, TkSQS2 and TkSQE1–4.** Alignments were created with Clustal MUSCLE (Edgar, 2004; http://www.ebi.ac.uk/Tools/msa/muscle/). Dashes represent gaps in aligned sequences, predicted transmembrane domains are underlined and conserved amino acids are shown in bold face. Protein sequences were obtained from GenBank (https://www.ncbi.nlm.nih.gov/genbank/). A: TkSQS1 and TkSQS2 amino acid alignment with SQS sequences from *Z. mays* (ZmSQS, NP_001104839.1), *A. thaliana* (AtSQS1, BAA06103.1), *A. annua* (AAR20329.1), *H. annuus* (OTG10658.1), *N. tabacum* (AAB08578.1) and *H. brasiliensis* (XP_021655633.1). Conserved sites include domains A (yellow, first step of catalysis), B (green, substrate binding) and C (blue, second step of catalysis). Putative hinge region is boxed. B: TkSQE1-4 amino acid alignment with SQE sequences from *A. thaliana* (AtSQE1, AAM20494.1), *H.* *annuus* (XP_021988698.1) and *P. ginseng* (PgSQE1, BAD15330.1; PgSQE2, ACJ24907.2). FAD ’fingerprint’ motif I (green) and FAD ‘fingerprint’ motif II (blue) are present.


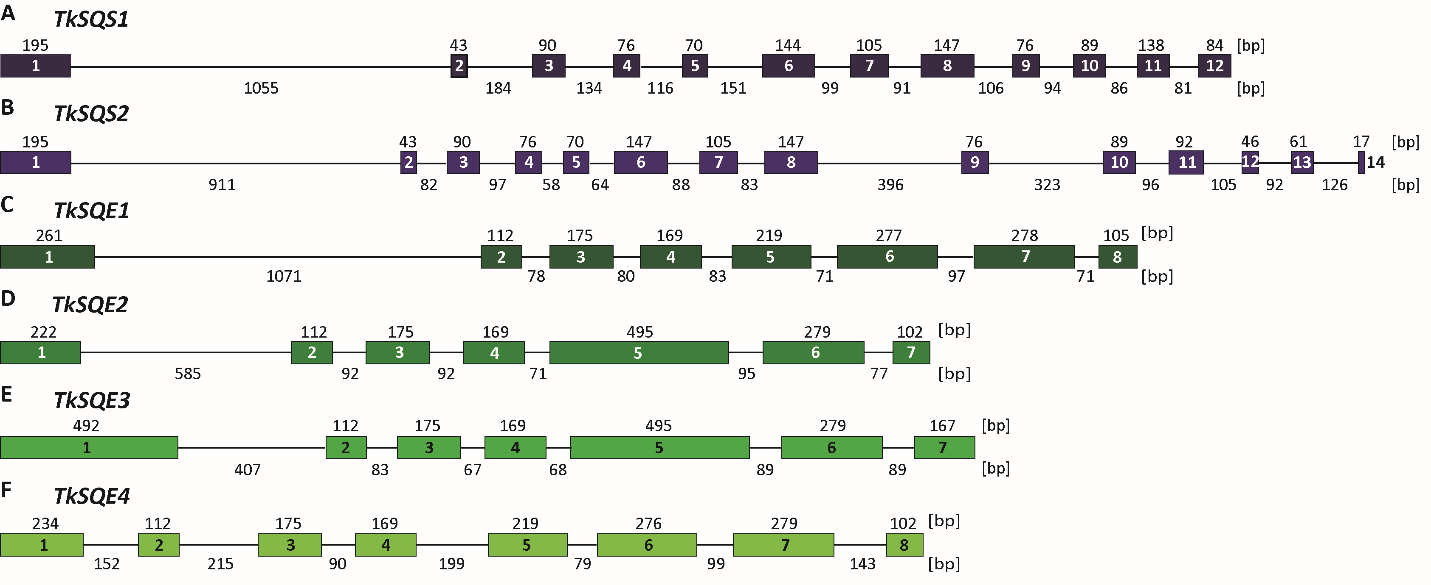


**Figure S2: Genomic map of the *TkSQS1*-*2* and *TkSQE1*-*4* loci.** Intron–exon structure of A: *TkSQS1*, B: *TkSQS2*, C: *TkSQE1*, D: *TkSQE2*, E: *TkSQE3* and F: *TkSQE4*, with exons represented by numbered boxes starting at the 5′ end of the genes, and introns represented by lines.


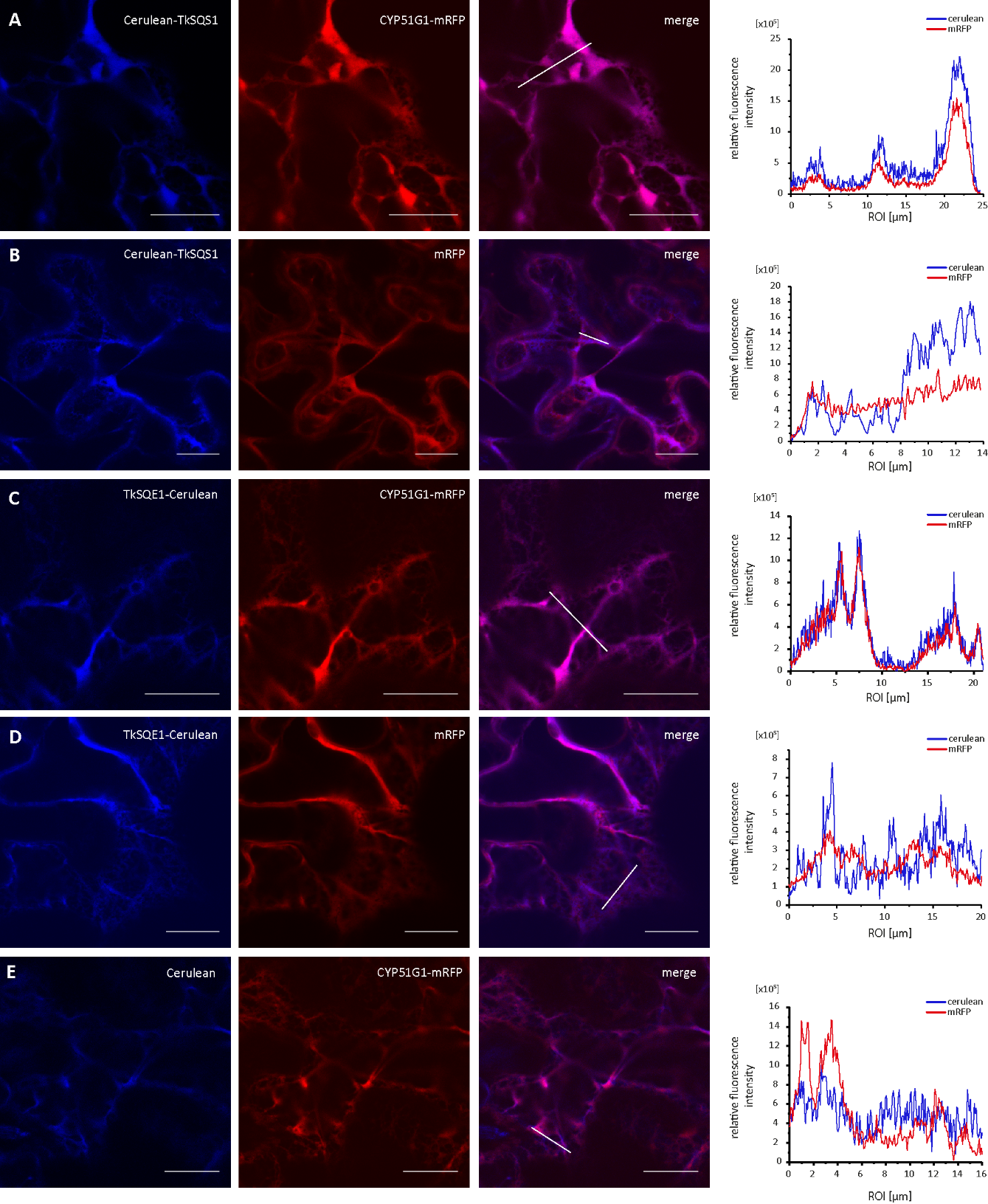


**Figure S3: Localization of TkSQS1 (A and B) and TkSQE1 (C and D) with focus on ER structures and Cerulean and CYP51G1-mRFP fluorescence (E) in *N. benthamiana* cells as a control for localization analysis of TkSQS1 and TkSQE1.** The CLSM images show red (mRFP, excitation 543 nm) and blue (Cerulean, excitation 458 nm) fluorescence of fusion proteins expressed in *N. benthamiana* epidermal cells. The relative fluorescence intensity in each region of interest (ROI) is marked with a white bar. NtermCYP51G1-mRFP = ER-localized marker containing a 120 bp cDNA fragment representing the N-terminus of *A. thaliana* CYP51G1 sterol 14-demethylase; mRFP = cytosolic marker; scale bar = 20 μm.


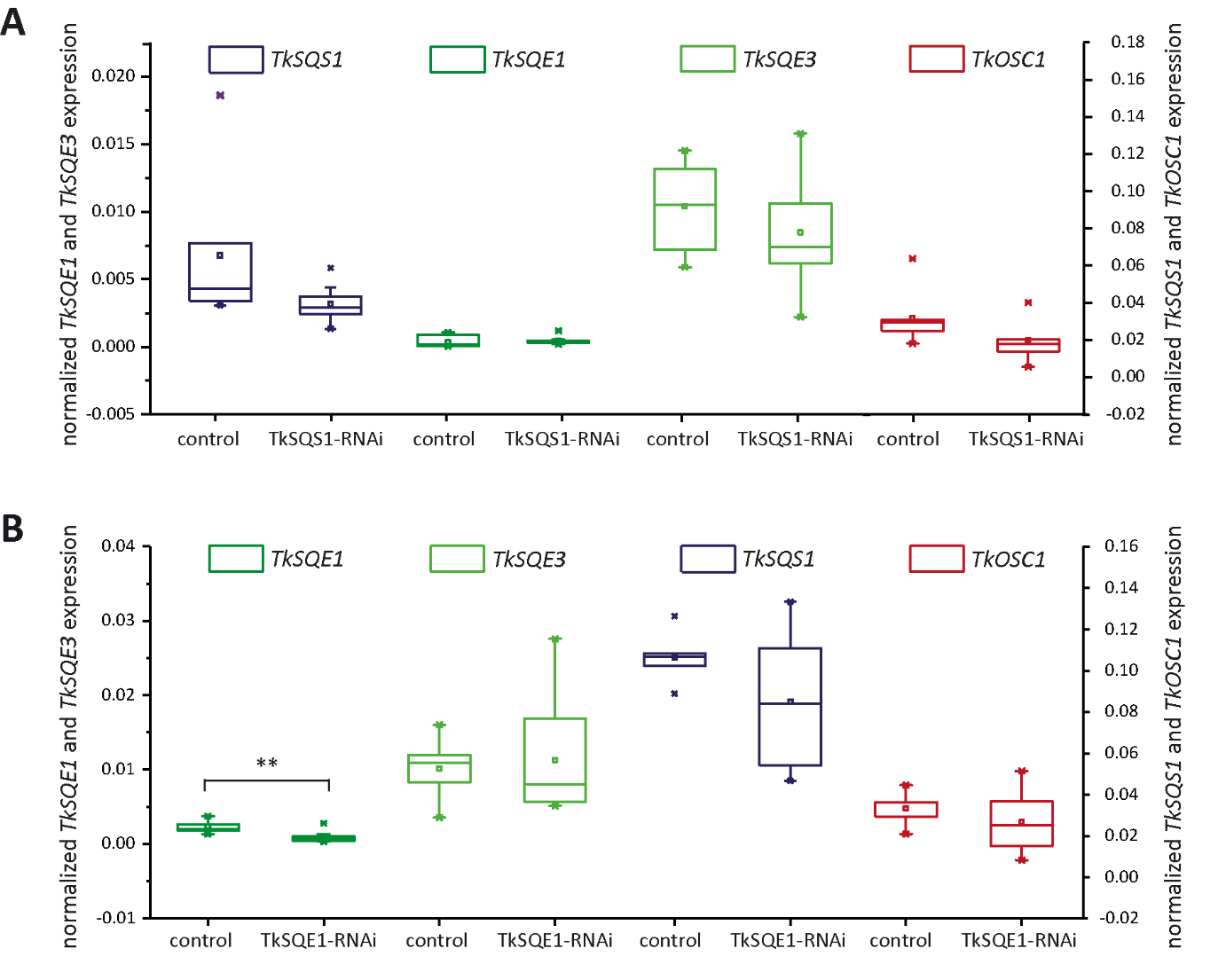


**Figure S4: *TkSQS1, TkSQE1, TkSQE3* and *TkOSC1* mRNA expression analysis in root material of transgenic TkSQS1-RNAi (A) and TkSQE1-RNAi (B) *T. koksaghyz* plants.** The corresponding mRNA levels were normalized against the constitutive genes encoding elongation factor 1α (*TkEF1α*) and ribosomal protein L27 (*TkRP*) from *T. koksaghyz*. The transgenic plants were analyzed 12 weeks after sowing. A: TkSQS1-RNAi plants (n=10 from 6 independent lines) compared to control plants (n=7) and B: TkSQE1-RNAi plants (n=10 from 4 independent lines) compared to control plants (n=5). Asterisks denote statistical significance compared to control (two-tailed t test, ** = p<0.01, *** = p<0.001).

**Table S1:** **Protein sequence data and corresponding accession numbers obtained from GenBank.**

| **Protein** | **Accession number** |
| --- | --- |
| **SQS proteins** | |
| *A. annua* SQS (AaSQS) | AAR20329.1 |
| *A. thaliana* SQS1 (AtSQS1) | BAA06103.1 |
| *B. platyphylla*SQS (BpSQS) | AKR76253.1 |
| *C. annuum* SQS (CaSQS) | AAD20626.1 |
| *C. cajan* SQS (CcSQS) | XP_020239442.1 |
| *C. melo* SQS (CmSQS) | XP_008461009.1 |
| *E. tirucalli* SQS (EtSQS) | BAH23428.1 |
| *G. glabra* SQS (GgSQS) | BAA13083.1 |
| *G. max* SQS1 (GmSQS1) | BAA22559.1 |
| *H. annuus* SQS (HaSQS) | OTG10658.1 |
| *H. brasiliensis* SQS (HbSQS) | XP_021655633.1 |
| *H. sapiens* SQS (HsSQS) | AAB33404.1 |
| *L. japonicus* SQS (LjSQS) | BAC56854.1 |
| *N. tabacum* SQS (NtSQS) | AAB08578.1 |
| *M. musculus* SQS (MmSQS) | NP_034321.2 |
| *M. truncatula* SQS (MtSQS) | XP_003607040.2 |
| *O. sativa* SQS (OsSQS) | BAA22557.1 |
| *P. ginseng* SQS1 (PgSQS1) | BAD08242.1 |
| *P. ginseng* SQS2 (PgSQS2) | ACV88718.1 |
| *P. ginseng* SQS3 (PgSQS3) | ACZ71037.1 |
| *S. cerevisiae* ERG9 (ScERG9) | AAA34597.1 |
| *S. indicum* SQS (SiSQS) | XP_011092839.1 |
| *S. tuberosum* SQS (StSQS) | BAA82093.1 |
| *T. koksaghyz* SQS1 (TkSQS1) | MG646369 |
| *T. koksaghyz* SQS2 (TkSQS2) | MG646370 |
| *W. somnifera* SQS (WsSQS) | ADC95435.1 |
| *Z. mays* SQS (ZmSQS) | NP_001104839.1 |
| **SQE proteins** | |
| *A. thaliana* SQE1 (AtSQE1) | AAM20494.1 |
| *A. thaliana* SQE2 (AtSQE2) | AEC07362.1 |
| *A. thaliana* SQE3 (AtSQE3) | Q8VYH2.1 |
| *B. platyphylla* SQE1 (BpSQE1) | AKR76254.1 |
| *C. borivilianum* SQE1 (CbSQE1) | AFN61200.1 |
| *C. melo* SQE1 (CmSQE1) | XP_016901349.1 |
| *E. tirucalli* SQE1 (EtSQE1) | BAF79915.1 |
| *G. max* SQE1 (GmSQE1) | XP_003529275.1 |
| *H. annuus* SQE1 (HaSQE1) | XP_021988698.1 |
| *H. brasiliensis* SQE1 (HbSQE1) | XP_021681283.1 |
| *H. sapiens* SQE1 (HsSQE1) | NP_003120.2 |
| *M. musculus* SQE1 (MmSQE1) | NP_033296.1 |
| *M. truncatula* SQE1 (MtSQE1) | CAD23249.1 |
| *M. truncatula* SQE2 (MtSQE2) | CAD23248.1 |
| *N. sativa* SQE1 (NsSQE1) | ACJ05633.1 |
| *O. sativa* SQE1 (OsSQE1) | AAO00687. |
| *P. ginseng* SQE1 (PgSQE1) | BAD15330.1 |
| *P. ginseng* SQE2 (PgSQE2) | ACJ24907.2 |
| *P. notoginseng* SQE1 (PnSQE1) | AGS79227.1 |
| *P. notoginseng* SQE2 (PnSQE2) | AFV92748.1 |
| *S. cerevisiae* ERG1 (ScERG1) | NP_011691.1 |
| *T. koksaghyz* SQE1 (TkSQE1) | MG646371 |
| *T. koksaghyz* SQE2 (TkSQE2) | MG646372 |
| *T. koksaghyz* SQE3 (TkSQE3) | MG646373 |
| *T. koksaghyz* SQE4 (TkSQE4) | MG646374 |
| *Z. mays* SQE1 (ZmSQE1) | ONL95392.1 |

**Table S2: Sequences of oligonucleotides used for cloning and qRT-PCR.**

| **Oligo** | **Sequence (5'→3')** |
| --- | --- |
| **full-length cDNA isolation** | |
| TkSQE1-fwd | ATGGAGCTTGGATTACCGCTG |
| TkSQE1-rev | TCACGCAACACGTGGAGAC |
| TkSQE2-fwd | ATGGCGGATCGCTTAATG |
| TkSQE2-rev | TTATGAAGGAGGAGC |
| TkSQE3-fwd | ATGGTTCCTATAAAACCCGCC |
| TkSQE3-rev | TTAAGATACCACACTATAC |
| TkSQE4-fwd | ATGGAGTTGAAGATATTACAAC |
| TkSQE4-rev | CTAAAAAGGCGATTTATAA |
| TkSQS1-fwd | ATGGGGAGTTTAAAGGCAGTAT |
| TkSQS1-rev | TCACAAAGTAACCTTGAATTTATTTGTGC |
| TkSQS2-fwd | ATGGGGACTTTAAAGGCGGTG |
| TkSQS2-rev | TCACGATGTAGCTTCTTTGGATTTG |
| **cloning procedures** | |
| Cerulean-BglII-fwd | AAAAGATCTATGGTTAGCAAAGGAGAAG |
| Cerulean-BglII-rev | AAAAGATCTTTATTTGTATAGTTCATCC |
| TkSQE1-fwd-NcoI | aaaccatggagcttggattaccg |
| TkSQE1-rev-NotI | AAAGCGGCCGCTCACGCAACACGTGGAGAC |
| TkSQE1-wos-rev-NotI | AAAGCGGCCGCCACGCAACACGTGGAGAC |
| TkSQE1-RNAi-fwd-NcoI | AAACCATGGCGAATAGTAGGTGAACTTC |
| TkSQE1-RNAi-rev-XhoI | AAACTCGAGGTCTTTCTCAAGCAATGAAG |
| TkSQS1-fwd-blunt | ATGGGGAGTTTAAAGGCAG |
| TkSQS1-rev-XhoI | AAACTCGAGTCACAAAGTAACCTTGAATTTAT |
| TkSQS1-RNAi-fwd-NcoI | AAACCATGGGGAGTTTAAAGGCAG |
| TkSQS1-RNAi-rev-XhoI | AAACTCGAGGATTGAGCTGTTGAATAACAAG |
| **qRT-PCR** | |
| TkEF1alpha-fw-realtime | CGAGAGATTCGAGAAGGAAGC |
| TkEF1alpha-rv-realtime | CTGTGCAGTAGTACTTGGTGG |
| TkOSC1-fw-realtime | ACTCCTCCCTTGATAATTGCCC |
| TkOSC1-rv-realtime | TTGTGCTTCTGCCTGATATATAGAAC |
| TkRP-fw-realtime | CGTCGATCTCAAGGATGTTGTC |
| TkRP-rv-realtime | GGAGCTTTGAGAAGAACCAACG |
| TkSQS1-fw-realtime | CTCAATGCTCAAGGCCAAGG |
| TkSQS1-rv-realtime | GCTTGGCTCGCTCTTAACAA |
| TkSQS2-fw-realtime | GATACGCGAGTAAACCAGAGGAT |
| TkSQS2-rv-realtime | AGGATCGCGTATGCCAGATAA |
| TkSQE1-fw-realtime | ATTTCTTCGCGGTGGCTATTTAC |
| TkSQE1-rv-realtime | CTTCCGACTTAATGATTGGGAAG |
| TkSQE2-fw-realtime | AAGCAAGGCAAGAGATGCGAGAAGC |
| TkSQE2-rv-realtlime | AAACACCGCGACTGCAAAAAAGTGC |
| TkSQE3-fw-realtime | CGCCGCCGTTGATCTCATCT |
| TkSQE3-rv-realtime | CGGCGCCGTAGAACCGATAG |
| TkSQE4-fw-realtime | ACAACCCACACCAGGAGCAA |
| TkSQE4-rv-realtime | GCAAAGGGCAATCGTGTCGT |

**Table S3:** **Primer efficiency and amplification factors for cDNA obtained from *T. koksaghyz* mRNA.** The values were calculated using the Bio-Rad CFX Manager v3.1 software (Bio-Rad Laboratories Inc., Hercules, CA, USA) and the qPCR primer efficiency calculator provided by Thermo Fisher Scientific (http://www.thermoscientificbio.com/webtools/qpcrefficiency/).

| **Oligo pair** | **Efficiency** | **Amplification factor (66°C)** |
| --- | --- | --- |
| TkOSC1-realtime | 100.16% | 2.00 |
| TkSQE1-realtime | 95.53% | 1.96 |
| TkSQE2-realtime | 116.05% | 2.16 |
| TkSQE3-realtime | 104.44% | 2.04 |
| TkSQE4-realtime | 98.64% | 1.99 |
| TkSQS1-realtime | 102.56% | 2.03 |
| TkSQS2-realtime | 104.76% | 2.05 |
| TkEF1alpha-realtime | 98.80% | 1.99 |
| TkRP-realtime | 97.20% | 1.97 |

**Table S4: Influence of MeJA treatment on gene expression of *TkSQS* and *TkSQE* genes in 8-week-old *T. koksaghyz* wild-type plants.** Six hours after treatment of 8-week-old *T. kokzaghyz* wild type plants (n=3) with MeJA or water as a control latex was harvested. The corresponding mRNA was used for qPCR analysis and normalized against the constitutive genes elongation factor 1 α (*TkEF1α*) and ribosomal protein L27 (*TkRP*). Two-tailed t test (p>0.01) revealed no significant differences between the expression of *TkSQS1-2* and *TkSQE1-4* in control and MeJA-treated plants.

|  | *TkSQS1* | *TkSQS2* | *TkSQE1* | *TkSQE2* | *TkSQE3* | *TkSQE4* |
| --- | --- | --- | --- | --- | --- | --- |
| **control** | 0.585  ± 0.250 | 0.000 | 0.023  ± 0.009 | 0.000 | 0.004  ± 0.001 | 0.000 |
| **MeJA treatment** | 0.791  ± 0.107 | 0.000 | 0.046  ± 0.013 | 0.000 | 0.010  ± 0.012 | 0.001  ± 0.001 |

**Table S5: Properties of identified *SQS* and *SQE* genes from *T. koksaghyz*.** aa, amino acids; bp, base pairs; pI, isoelectric point; kDa, kilo Dalton; Mw, molecular weight; TMD, transmembrane domain.

|  | **gDNA length [bp]** | **No. of exons** | **cDNA length [bp]** | **protein size [aa]** | **Mw [kDa]** | **pI** | **predicted TMDs** | **accession number** |
| --- | --- | --- | --- | --- | --- | --- | --- | --- |
| **TkSQS1** | 3,454 | 12 | 1,257 | 418 | 47.981 | 8.18 | 1 | MG646369 |
| **TkSQS2** | 3,775 | 14 | 1,254 | 417 | 47.713 | 8.57 | 1 | MG646370 |
| **TkSQE1** | 3,146 | 8 | 1,596 | 513 | 58.080 | 8.81 | 4 | MG646371 |
| **TkSQE2** | 2,566 | 7 | 1,554 | 517 | 56.399 | 8.93 | 1 | MG646372 |
| **TkSQE3** | 2,692 | 7 | 1,857 | 618 | 68.107 | 9.39 | 6 | MG646373 |
| **TkSQE4** | 2,543 | 8 | 1,566 | 521 | 56.550 | 8.88 | 2 | MG646374 |

Table S6: Temporal changes in the levels of sterol precursors, sterols and pentacyclic triterpenes in the roots of *T. kokzaghyz* wild-type plants ranging from 8 to 20 weeks. Single triterpene compounds were identified and quantified by GC/MS [mg/g root dry weight] and corresponding retention indices (RI) were determined in relation to a C8–C40 alkane calibration standard. The mean values represent independent extractions of three (* = two) pools of three wild-type plants each (n = 3).

|  | **RI** | **8 weeks*** | **10 weeks** | **12 weeks*** | **14 weeks** | **16 weeks** | **18 weeks** | **20 weeks** |
| --- | --- | --- | --- | --- | --- | --- | --- | --- |
| **sterol precursors** |  |  |  |  |  |  |  |  |
| squalene | 2843 | 0.08±0.03 | 0.24±0.14 | 0.22±0.01 | 0.13±0.01 | 0.15±0.03 | 0.14±0.02 | 0.13±0.03 |
| 2,3-oxidosqualene | 2965 | 0.08±0.02 | 0.26±0.04 | 0.33±0.04 | 0.40±0.08 | 0.54±0.10 | 0.33±0.07 | 0.43±0.11 |
| **sterols** |  |  |  |  |  |  |  |  |
| campesterol | 3313 | 0.32±0.01 | 0.40±0.05 | 0.31±0.04 | 0.28±0.01 | 0.29±0.03 | 0.30±0.06 | 0.27±0.04 |
| stigmasterol | 3343 | 0.75±0.02 | 1.04±0.04 | 0.78±0.03 | 0.63±0.05 | 0.64±0.11 | 0.61±0.07 | 0.62±0.07 |
| sitosterol | 3405 | 1.12±0.05 | 1.28±0.06 | 1.19±0.02 | 0.81±0.07 | 0.94±0.13 | 0.88±0.12 | 0.84±0.08 |
| **pentacyclic triterpenes** |  |  |  |  |  |  |  |  |
| taraxerol and unknown triterpene | 3448 | 1.34±0.12 | 1.49±0.17 | 1.51±0.24 | 1.43±0.16 | 1.62±0.10 | 1.50±0.21 | 1.66±0.08 |
| ß-amyrin | 3465 | 8.83±0.83 | 7.56±0.83 | 5.84±1.20 | 4.50±0.65 | 4.48±0.62 | 4.43±0.77 | 4.48±0.46 |
| lupeol | 3510 | 0.23±0.12 | 0.20±0.01 | 0.15±0.02 | 0.12±0.00 | 0.12±0.01 | 0.11±0.02 | 0.12±0.00 |
| α-amyrin | 3516 | 5.57±0.31 | 4.58±0.56 | 3.32±0.75 | 2.67±0.31 | 2.59±0.27 | 2.79±0.56 | 2.74±0.31 |
| unknown triterpenes | 3590 | 0.36±0.00 | 0.44±0.01 | 0.48±0.07 | 0.46±0.10 | 0.51±0.06 | 0.45±0.06 | 0.55±0.06 |
| unknown triterpenes | 3603 | 2.52±0.25 | 2.06±0.23 | 1.59±0.35 | 1.21±0.20 | 1.22±0.15 | 1.21±0.22 | 1.18±0.14 |
| taraxasterol | 3615 | 9.25±0.98 | 7.73±0.90 | 5.84±1.17 | 4.68±0.71 | 4.67±0.52 | 4.68±0.82 | 4.44±0.30 |

**Table S7: Levels of sterol precursors, sterols and pentacyclic triterpenes in root material from TkSQS1-RNAi lines (n=10) and the corresponding control plants (n=7).** Single triterpene compounds were identified and quantified by GC/MS [mg/g root dry weight] and corresponding retention indices (RI) were determined in relation to a C8–C40 alkane calibration standard. The mean values represent three independent extractions.

|  | **RI** | **1A** | **1B** | **2A** | **2C** | **4** | **5** |
| --- | --- | --- | --- | --- | --- | --- | --- |
| **sterol precursors** |  |  |  |  |  |  |  |
| squalene | 2843 | 0.12±0.01 | 0.18±0.04 | 0.09±0.01 | 0.27±0.03 | 0.10±0.04 | 0.08±0.01 |
| 2,3-oxidosqualene | 2965 | 0.38±0.03 | 0.80±0.00 | 0.31±0.02 | 0.23±0.01 | 0.18±0.01 | 0.26±0.01 |
| **sterols** |  |  |  |  |  |  |  |
| campesterol | 3313 | 0.16±0.00 | 0.16±0.01 | 0.16±0.01 | 0.26±0.01 | 0.17±0.01 | 0.27±0.01 |
| stigmasterol | 3343 | 0.37±0.01 | 0.40±0.01 | 0.45±0.02 | 0.76±0.04 | 0.40±0.03 | 0.70±0.01 |
| sitosterol | 3405 | 0.51±0.04 | 0.50±0.01 | 0.45±0.02 | 0.51±0.01 | 0.49±0.01 | 0.45±0.01 |
| **pentacyclic triterpenes** |  |  |  |  |  |  |  |
| taraxerol and unknown triterpene | 3448 | 1.06±0.02 | 1.11±0.02 | 0.75±0.01 | 0.64±0.02 | 0.81±0.03 | 0.69±0.02 |
| ß-amyrin | 3465 | 1.94±0.10 | 2.50±0.07 | 0.95±0.01 | 1.21±0.11 | 1.53±0.04 | 2.53±0.17 |
| lupeol | 3510 | 0.14±0.01 | 0.25±0.01 | 0.06±0.01 | 0.07±0.01 | 0.05±0.02 | 0.15±0.01 |
| α-amyrin | 3516 | 1.25±0.03 | 1.62±0.03 | 0.52±0.01 | 0.60±0.01 | 0.85±0.03 | 1.38±0.08 |
| unknown triterpenes | 3590 | 0.36±0.01 | 0.58±0.01 | 0.41±0.02 | 0.31±0.02 | 0.29±0.01 | 0.34±0.01 |
| unknown triterpenes | 3603 | 0.79±0.02 | 0.95±0.01 | 0.42±0.01 | 0.46±0.01 | 0.61±0.03 | 1.00±0.01 |
| taraxasterol | 3615 | 2.26±0.08 | 2.77±0.03 | 1.18±0.05 | 1.49±0.05 | 2.12±0.07 | 2.99±0.09 |

|  | **RI** | **6B** | **6D** | **10A** | **10B** |
| --- | --- | --- | --- | --- | --- |
| **sterol precursors** |  |  |  |  |  |
| squalene | 2843 | 0.23±0.01 | 0.08±0.01 | 0.07±0.01 | 0.09±0.01 |
| 2,3-oxidosqualene | 2965 | 0.34±0.01 | 0.52±0.01 | 0.19±0.01 | 0.41±0.02 |
| **sterols** |  |  |  |  |  |
| campesterol | 3313 | 0.17±0.01 | 0.19±0.01 | 0.16±0.01 | 0.16±0.01 |
| stigmasterol | 3343 | 0.46±0.01 | 0.48±0.01 | 0.30±0.01 | 0.36±0.01 |
| sitosterol | 3405 | 0.43±0.04 | 0.48±0.01 | 0.38±0.01 | 0.45±0.03 |
| **pentacyclic triterpenes** |  |  |  |  |  |
| taraxerol and unknown triterpene | 3448 | 0.55±0.02 | 0.65±0.03 | 0.67±0.03 | 0.76±0.01 |
| ß-amyrin | 3465 | 0.96±0.02 | 2.23±0.03 | 0.71±0.01 | 1.47±0.02 |
| lupeol | 3510 | 0.05±0.01 | 0.08±0.02 | 0.04±0.01 | 0.06±0.01 |
| α-amyrin | 3516 | 0.56±0.02 | 1.37±0.11 | 0.40±0.01 | 0.82±0.01 |
| unknown triterpenes | 3590 | 0.19±0.01 | 0.19±0.01 | 0.32±0.01 | 0.23±0.01 |
| unknown triterpenes | 3603 | 0.36±0.01 | 1.81±0.01 | 0.28±0.02 | 0.58±0.02 |
| taraxasterol | 3615 | 1.18±0.02 | 2.56±0.05 | 0.92±0.01 | 1.77±0.03 |

|  | **RI** | **Control 1** | **Control 2** | **Control 3** | **Control 4** | **Control 5** | **Control 6** | **Control 7** |
| --- | --- | --- | --- | --- | --- | --- | --- | --- |
| **sterol precursors** |  |  |  |  |  |  |  |  |
| squalene | 2843 | 0.15±0.01 | 0.07±0.02 | 0.14±0.01 | 0.79±0.03 | 0.11±0.02 | 0.08±0.01 | 0.06±0.02 |
| 2.3-oxidosqualene | 2965 | 0.61±0.02 | 0.25±0.02 | 0.50±0.03 | 0.48±0.06 | 0.25±0.02 | 0.52±0.01 | 0.11±0.01 |
| **sterols** |  |  |  |  |  |  |  |  |
| campesterol | 3313 | 0.18±0.01 | 0.14±0.01 | 0.18±0.01 | 0.20±0.01 | 0.17±0.01 | 0.19±0.02 | 0.14±0.01 |
| stigmasterol | 3343 | 0.39±0.01 | 0.28±0.01 | 0.37±0.02 | 0.48±0.02 | 0.34±0.02 | 0.48±0.01 | 0.31±0.01 |
| sitosterol | 3405 | 0.42±0.01 | 0.44±0.02 | 0.43±0.01 | 0.49±0.02 | 0.49±0.02 | 0.48±0.03 | 0.46±0.02 |
| **pentacyclic triterpenes** |  |  |  |  |  |  |  |  |
| taraxerol and unknown triterpene | 3448 | 0.77±0.02 | 0.67±0.01 | 0.65±0.02 | 0.71±0.01 | 0.79±0.01 | 0.65±0.01 | 0.69±0.04 |
| ß-amyrin | 3465 | 1.19±0.07 | 0.76±0.04 | 1.54±0.07 | 2.00±0.06 | 1.49±0.04 | 2.23±0.10 | 1.49±0.04 |
| lupeol | 3510 | 0.05±0.01 | 0.05±0.01 | 0.05±0.01 | 0.07±0.02 | 0.20±0.01 | 0.08±0.01 | 0.07±0.02 |
| α-amyrin | 3516 | 0.69±0.03 | 0.45±0.01 | 0.90±0.01 | 1.06±0.06 | 1.03±0.04 | 1.37±0.03 | 0.90±0.01 |
| unknown triterpenes | 3590 | 0.36±0.01 | 0.20±0.01 | 0.24±0.01 | 0.12±0.01 | 0.35±0.01 | 0.19±0.01 | 0.34±0.01 |
| unknown triterpenes | 3603 | 0.45±0.01 | 0.34±0.01 | 0.59±0.01 | 0.68±0.02 | 0.59±0.03 | 0.81±0.01 | 0.61±0.01 |
| taraxasterol | 3615 | 1.42±0.03 | 1.07±0.01 | 1.89±0.05 | 2.07±0.01 | 1.82±0.04 | 2.56±0.01 | 1.80±0.06 |

**Table S8: Levels of sterol precursors. sterols and pentacyclic triterpenes in root material from TkSQE1-RNAi lines (n=11) and the corresponding control plants (n=6).** Single triterpene compounds were identified and quantified by GC/MS [mg/g root dry weight] and corresponding retention indices (RI) were determined in relation to a C8–C40 alkane calibration standard. The mean values represent three independent extractions.

|  | **RI** | **A.1** | **A.2** | **B.1** | **C.1** | **C.2** |
| --- | --- | --- | --- | --- | --- | --- |
| **sterol precursors** |  |  |  |  |  |  |
| squalene | 2843 | 9.77±0.19 | 14.36±1.04 | 9.16±0.19 | 6.01±0.26 | 14.43±0.91 |
| 2.3-oxidosqualene | 2965 | 0.95±0.04 | 0.75±0.07 | 0.68±0.09 | 0.59±0.04 | 1.23±0.07 |
| **sterols** |  |  |  |  |  |  |
| campesterol | 3313 | 0.51±0.06 | 0.48±0.02 | 0.72±0.04 | 0.69±0.02 | 0.47±0.01 |
| stigmasterol | 3343 | 1.07±0.04 | 0.98±0.07 | 1.18±0.05 | 0.94±0.01 | 1.05±0.03 |
| sitosterol | 3405 | 2.13±0.18 | 1.45±0.14 | 1.77±0.1 | 1.52±0.12 | 1.61±0.07 |
| **pentacyclic triterpenes** |  |  |  |  |  |  |
| taraxerol and unknown triterpene | 3448 | 1.36±0.05 | 1.26±0.09 | 1.43±0.09 | 1.35±0.05 | 2.66±0.03 |
| ß-amyrin | 3465 | 1.69±0.04 | 1.75±0.01 | 2.93±0.03 | 1.70±0.06 | 2.25±0.12 |
| lupeol | 3510 | 0.10±0.02 | 0.12±0.02 | 0.10±0.02 | 0.19±0.04 | 0.08±0.03 |
| α-amyrin | 3516 | 1.00±0.04 | 1.06±0.04 | 1.73±0.07 | 0.99±0.05 | 1.36±0.05 |
| unknown triterpenes | 3590 | 0.33±0.05 | 0.55±0.04 | 0.44±0.02 | 0.37±0.02 | 0.83±0.02 |
| unknown triterpenes | 3603 | 0.54±0.01 | 0.59±0.03 | 0.98±0.02 | 0.65±0.02 | 0.85±0.02 |
| taraxasterol | 3615 | 1.93±0.04 | 2.27±0.12 | 3.81±0.10 | 2.30±0.11 | 2.99±0.07 |

|  | **RI** | **D.1** | **D.2** | **D.3** | **D.4** | **D.5** | **D.6** |
| --- | --- | --- | --- | --- | --- | --- | --- |
| **sterol precursors** |  |  |  |  |  |  |  |
| squalene | 2843 | 0.47±0.04 | 8.03±0.25 | 1.36±0.09 | 1.30±0.12 | 4.08±0.28 | 9.42±1.29 |
| 2.3-oxidosqualene | 2965 | 2.36±0.15 | 1.77±0.10 | 2.36±0.13 | 1.37±0.08 | 1.06±0.05 | 0.89±0.06 |
| **sterols** |  |  |  |  |  |  |  |
| campesterol | 3313 | 0.70±0.02 | 0.46±0.07 | 0.64±0.05 | 0.64±0.03 | 0.88±0.06 | 0.60±0.04 |
| stigmasterol | 3343 | 1.05±0.07 | 0.92±0.03 | 1.04±0.02 | 0.89±0.03 | 1.41±0.04 | 0.93±0.07 |
| sitosterol | 3405 | 1.65±0.07 | 1.45±0.05 | 1.74±0.14 | 1.81±0.11 | 2.09±0.09 | 2.26±0.09 |
| **pentacyclic triterpenes** |  |  |  |  |  |  |  |
| taraxerol and unknown triterpene | 3448 | 1.40±0.04 | 1.04±0.04 | 1.31±0.04 | 1.55±0.03 | 1.56±0.03 | 1.82±0.12 |
| ß-amyrin | 3465 | 1.23±0.21 | 1.70±0.09 | 1.39±0.04 | 2.43±0.07 | 1.56±0.12 | 3.30±0.21 |
| lupeol | 3510 | 0.10±0.02 | 0.10±0.04 | 0.08±0.02 | 0.12±0.05 | 0.11±0.02 | 0.21±0.04 |
| α-amyrin | 3516 | 0.79±0.13 | 1.10±0.06 | 0.86±0.02 | 1.64±0.03 | 0.88±0.09 | 2.00±0.12 |
| unknown triterpenes | 3590 | 0.51±0.01 | 0.65±0.02 | 0.67±0.03 | 0.22±0.01 | 0.68±0.05 | 0.51±0.03 |
| unknown triterpenes | 3603 | 0.44±0.05 | 0.62±0.04 | 0.55±0.03 | 0.86±0.03 | 0.64±0.01 | 1.32±0.13 |
| taraxasterol | 3615 | 1.55±0.25 | 2.28±0.02 | 1.86±0.02 | 3.16±0.12 | 2.28±0.12 | 4.90±0.35 |

|  | RI | **Control 1** | **Control 2** | **Control 3** | **Control 4** | **Control 5** | **Control 6** |
| --- | --- | --- | --- | --- | --- | --- | --- |
| **sterol precursors** |  |  |  |  |  |  |  |
| squalene | 2843 | 0.39±0.06 | 0.20±0.03 | 0.33±0.04 | 0.17±0.03 | 0.34±0.09 | 0.22±0.00 |
| 2.3-oxidosqualene | 2965 | 0.92±0.06 | 0.51±0.07 | 0.54±0.08 | 0.48±0.01 | 0.99±0.22 | 0.70±0.09 |
| **sterols** |  |  |  |  |  |  |  |
| campesterol | 3313 | 0.95±0.04 | 0.53±0.02 | 0.87±0.05 | 0.78±0.04 | 0.60±0.10 | 0.52±0.03 |
| stigmasterol | 3343 | 1.19±0.02 | 0.85±0.01 | 1.13±0.11 | 0.96±0.04 | 1.36±0.41 | 0.85±0.02 |
| sitosterol | 3405 | 2.00±0.08 | 1.71±0.09 | 1.89±0.25 | 1.60±0.10 | 2.29±0.43 | 1.47±0.06 |
| **pentacyclic triterpenes** |  |  |  |  |  |  |  |
| taraxerol and unknown triterpene | 3448 | 1.90±0.10 | 1.44±0.03 | 2.24±0.23 | 1.67±0.05 | 2.80±0.74 | 1.79±0.05 |
| ß-amyrin | 3465 | 2.37±0.06 | 1.58±0.01 | 4.20±0.35 | 3.17±0.14 | 3.04±0.80 | 1.73±0.01 |
| lupeol | 3510 | 0.15±0.01 | 0.12±0.04 | 0.27±0.07 | 0.23±0.04 | 0.27±0.06 | 0.10±0.03 |
| α-amyrin | 3516 | 1.28±0.09 | 0.88±0.03 | 2.55±0.17 | 1.85±0.11 | 2.04±0.52 | 1.02±0.06 |
| unknown triterpenes | 3590 | 0.38±0.01 | 0.21±0.01 | 0.29±0.02 | 0.33±0.05 | 0.81±0.26 | 0.59±0.01 |
| unknown triterpenes | 3603 | 0.88±0.05 | 0.49±0.02 | 1.50±0.15 | 1.22±0.09 | 1.20±0.31 | 0.67±0.04 |
| taraxasterol | 3615 | 3.25±0.08 | 1.86±0.09 | 5.51±0.41 | 4.72±0.30 | 3.82±1.13 | 2.26±0.08 |
