# Supplementary material for: Functional characterization of squalene synthase and squalene epoxidase in Taraxacum koksaghyz
Source: Plant Direct. 2018 Jun 13;2(6):e00063. doi: 10.1002/pld3.63 (PMC6508512; doi:10.1002/pld3.63)
Supplement: Supplementary file 2 [file PLD3-2-e00063-s002.pdf]

Dear Feng Chen,

We would like to thank you for the opportunity to submit a revised version of our manuscript "Functional characterization of squalene synthase and squalene epoxidase in Taraxacum koksaghyz" (reference number Plant Direct: 2018-00120-T) and we very much appreciate the comments from yourself and the reviewer, which improved the presentation and interpretation of our data. We apologize for the time needed to revise the manuscript but you may recall that the reviewers requested a number of additional experiments, which we have now completed.

We have revised the manuscript according the comments and provide a point-by-point response below.

We hope the manuscript is now suitable for publication in *Plant Direct*.

Yours sincerely

Christian Schulze Gronover, on behalf of the co-authors

Von: amajoe@msubmit.net <amajoe@msubmit.net>
Gesendet: Freitag, 23. Februar 2018 19:29:32
An: Christian Schulze Gronover
Cc: PlantDirect@wiley.com
Betreff: Plant Direct: 2018-00120-T: Decision Letter

February 23, 2018

Dr. Christian Schulze Gronover
Fraunhofer Institute for Molecular Biology and Applied Ecology
Schlossplatz 8
Muenster 48143
Germany

RE: Functional characterization of squalene synthase and squalene epoxidase in Taraxacum koksaghyz

Dear Dr. Schulze Gronover:

Thank you for submitting to Plant Direct. All required reviews have been returned and we have now finished our evaluation of your manuscript. In light of the reviewers' comments, minor revisions are needed before the paper can be accepted for publication in Plant Direct.

Please view the editors' and reviewers' comments below and use their suggestions as a guide while you work on your revision.

When uploading the revised version of this article, please be sure to include the following:

-A word document that contains your response to the reviewers. You should respond to each

reviewer comment and note the changes made to the manuscript. If you do not agree with a reviewer's comment and choose not to make a suggested revision, please explain why. Please try to provide as complete an answer as possible to each reviewer's criticisms.
-A tracked changes document with each change highlighted
- A clean version of the latest version of the manuscript

Thank you very much for giving us an opportunity to review your work. I look forward to receiving the next version.

Sincerely,

Feng Chen

Editor, Plant Direct

-- Reviewer comments:
Reviewer #1 :

The manuscript contributed by Unland et al. reports on the cloning and functional characterization of squalene synthase and squalene epoxidase in the latex-producing plant Taraxacum koksaghyz. The manuscript focuses on the characterization of the latex-predominat isoforms TkSQS1 and TkSQE1, including confirmation of their enzyme activity, subcellular localization studies, detailed temporal and spatial gene expression and isoprenoid content profiles, and analysis of the effects of RNAi-mediated depletion of these isoforms on sterol and triterpene levels. These two last aspects are the most relevant contribution of the manuscript, despite depletion of TkSQS1 or TkSQE1 mRNA levels had no significant effects on isoprenoid biosynthesis contrary to what could have been expected. Nevertheless, this does not compromise at all the quality of work, although it highlights the growing need to approach the regulation of isoprenoid metabolism from a broader perspective including the different regulatory mechanisms operating at post-transcriptional level, a field of study that is still far behind the regulatory studies at the transcriptional level. In any case, the paper is nicely written, the experiments have been conducted in a rigorous and consistent manner, the results are presented in clear and intelligible form, well discussed, and the conclusions drawn are fully supported by the results. Overall, the manuscript is a sound piece of work that addresses a topic of interest.
I just have a couple of very minor comments. The reticulated pattern indicative of localization in the ER is not visible, at least in the images to which I have access. Is it possible to include a set of images showing it?
Authors' comment: We performed additional CLSM studies using the same constructs and conditions and included a set of new images showing the reticulated pattern of the ER and co-localization of the analyzed proteins at the ER in Figure S3.

The genomic map of TkSQS1 and TkSQE1 loci should be relegated to the supplemental material.

Authors' comment: We relegated the genomic map of TkSQS1-2 and TkSQE1-4 to the supplemental material (Figure S2).

Reviewer #2 :

General comments:
In this manuscript the authors present the functional characterization of SQS and SQE from Taraxacum koksaghyz. The study focuses on determining the biosynthesis of pentacyclic triterpenes and clearly elucidate the correlation of the key rate-limiting enzymes. As the predominate gene expressed in latex, SQS1 and SQE1 were carried out for the functional assay. In my opinion the data of this paper are generally presented quite clearly. I have only relatively minor comments about the results and data presentation.
(1) This study highlights the SQS1 and SQE1 expressed higher in latex, but why most of the triterpene measurement are conducted in root? Please explain the correlation of the SQS/SQE function and triterpene production in latex and root?
Authors' comment: Freeze-dried roots were used for triterpene extraction and GC-MS analysis because quantitative analysis of triterpenes in latex was not feasible for comparative analysis. *TkSQS1* and *TkSQE1* are the genes which are expressed highest in latex and roots and therefore account for most of the triterpenes produced in latex and root. Moreover, latex is highly abundant in roots. Accordingly, measuring the triterpene content in root material is the closest approximation to triterpene content in latex.

(2) I think the results part is too long and quite a large part should be addressed in methods part or discussion part.
Authors' comment: We revised the manuscript as requested and shortened the results part considerably as recommended.

(3) Some of the debate about non-effectiveness on the pentacyclic triterpenes content by the RNAi is a bit unconvincing.
Authors' comment: We tried to improve the debate by adding several additional experiments and discussing them (Figure S4).

(4) Although the expression of SQS2 and SQE2-4 are weak in the latex under the normal condition, I also wonder whether they can be induced by some specific biotic factors, like the treatment of MeJA or other hormones, which may indicate the role of these isoforms on the defense of other biological functions.
Authors' comment: We performed a MeJA treatment with 8-week-old *T. koksaghyz* wild-type plants (according to Cao et al., 2017; Table S4) and could see slightly elevated gene expression of *TkSQS1* and *TkSQE1* in *T. koksaghyz* latex. This might indicate a role in defense as has been reported for other *T. koksaghyz* genes involved in secondary metabolism (Cao et al., 2017; included in the discussion part of the manuscript). Expression of *TkSQS2* and *TkSQE2-4* was not or only hardly detectable after MeJA treatment indicating no predominant role of these isoforms in similar biological functions, but their role and especially their interplay with *TkSQS1* and *TkSQE1* should be investigated in more detail in future studies.

Specific comments:
Line 36-39 This sentence is somewhat confusing, please rephrase

Authors' comment: We rephrased the sentence as recommended.

Line 61-63 This sentence is somewhat confusing, please rephrase
Authors' comment: We rephrased the sentence as recommended.

Line 191-195 Why only the exon-intron structure of SQS1 and SQE1 were determined?
Authors' comment: We determined the exon-intron structures of *TkSQS1-2* and *TkSQE1-4* (Fig. S2).

Line 209 From Figure 4B, the expression of SQE3 is much higher than SQE 1 in root, why can it be excluded from the further investigation of the function, especially for involving the triterpene production in root?
Authors' comment: In latex, *TkSQE3* expression is very low and therefore not in focus of our RNAi approach. As we investigate triterpene biosynthesis in *T. koksaghyz* latex, only downregulation of the latex-predominant TkSQE1 should be achieved. Moreover, we also analyzed the gene expression in root material of TkSQ1-RNAi and TkSQE1-RNAi transgenic lines and could confirm that gene expression in root tissue is similar to gene expression in latex (Fig. S4A and B). Thus, we conclude that no other genes compensate for the respective reduced *TkSQS1* and *TkSQE1* expression. Accordingly, the other TkSQE isoforms were excluded from further investigations.

Line 220-223 better to put this sentence in discussion part.
Authors' comment: We revised the sentence and transferred it to the discussion part.

Line 227 "in" to "at"
Authors' comment: We would like to keep the statement as it is as we are referring to squalene content in plants.

Line 230-232 better to change the statement
Authors' comment: We changed the statement as recommended.

Line 240-249 better to state this in methods not in results
Authors' comment: We transferred this section to the method part as recommended.

Line 262-264 better to state this in discussion part
Authors' comment: We have revised the manuscript as recommended.

Line 265-271 better to state this in method part
Authors' comment: We transferred this section to the method part as recommended.

Line 327-330 better to state this in discussion part
Authors' comment: We transferred this section to the discussion part as recommended.

Line 337-339 better to change the statement
Authors' comment: We would like to keep the statement, as similar observations have been made for *G. glabra* isoforms that cluster on different branches of phylogenetic trees (Navarro Gallón et al., 2017). (Lines 432-434)

Line 430 better to change the statement
Authors’ comment: We changed the statement as recommended.

Line 454 In this part, please also describe in detail how were SQS and SQE sequences were identified from draft genome sequence or RNA-Seq data.
Authors’ comment: We described the identification of the *SQS* and *SQE* sequences as recommended.

Line 493 20 klux seems be too low for the growth.
Authors’ comment: We do have specialized 20 klux high pressure sodium lamps, HPS 600 Watts, Greenbud, with an enhanced yellow and red spectrum to ensure optimal growth.

Line 545 better to describe the steps of triterpene collection and identification a bit more in detail.
Authors’ comment: We revised the manuscript as recommended.

Line 585 Is this reference from a journal or book?
Authors’ comment: Thank you for the notification. This reference is from a book and has been edited accordingly in the bibliography.

Line 589 species name should be Italic, please check through all the MS.
Authors’ comment: Species names were revised as recommended.

Figures and Tables:
Figure 4: The content of Squalene and 2,3-oxidosqualene in Figure 4D and Sterol and pentacyclic triterpene in Figure 4F are identified from latex or root?
Authors’ comment: The corresponding contents were identified from roots. We revised the manuscript as recommended.

Table 3S: If possible, it's better to add RI for the identification of specific compounds.
Authors’ comment: We added the RI for the identification of specific compounds and revised the manuscript as recommended.

Table 6S and Table 7S： Why there are replicates of the measurement in Table 7S, but not in Table 6S?
Authors’ comment: We measured the root material depicted in Table S6 in triplicates and added the deviation as well as the RI for each compound.
